# Carbon Nanotubes: Current Perspectives on Diverse Applications in Targeted Drug Delivery and Therapies

**DOI:** 10.3390/ma14216707

**Published:** 2021-11-07

**Authors:** Mohamed Rahamathulla, Rohit R. Bhosale, Riyaz A. M. Osmani, Kasturi C. Mahima, Asha P. Johnson, Umme Hani, Mohammed Ghazwani, Mohammed Y. Begum, Sultan Alshehri, Mohammed M. Ghoneim, Faiyaz Shakeel, Hosahalli V. Gangadharappa

**Affiliations:** 1 Department of Pharmaceutics, College of Pharmacy, King Khalid University, Abha 61421, Saudi Arabia; shmohamed@kku.edu.sa (M.R.); ummehaniahmed@gmail.com (U.H.); myghazwani@kku.edu.sa (M.G.); ybajen@kku.edu.sa (M.Y.B.); 2 Department of Pharmaceutics, Krishna Institute of Pharmacy, Krishna Institute of Medical Sciences “Deemed To Be University”, Karad 415539, Maharashtra, India; bhosalerohit707@gmail.com; 3 Department of Pharmaceutics, JSS College of Pharmacy, JSS Academy of Higher Education and Research, Mysuru 570015, Karnataka, India; riyazosmani@gmail.com (R.A.M.O.); mahimakasturic@gmail.com (K.C.M.); ashapjohnson19@gmail.com (A.P.J.); 4 Department of Pharmaceutics, College of Pharmacy, King Saud University, Riyadh 11451, Saudi Arabia; salshehri1@ksu.edu.sa (S.A.); faiyazs@fastmail.fm (F.S.); 5 Department of Pharmacy Practice, College of Pharmacy, AlMaarefa University, Ad Diriyah 13713, Saudi Arabia; mghoneim@mcst.edu.sa

**Keywords:** carbon nanotubes, toxicity, targeted drug delivery, therapies, biomedical applications, patents

## Abstract

Current discoveries as well as research findings on various types of carbon nanostructures have inspired research into their utilization in a number of fields. These carbon nanostructures offer uses in pharmacy, medicine and different therapies. One such unique carbon nanostructure includes carbon nanotubes (CNTs), which are one-dimensional allotropes of carbon nanostructure that can have a length-to-diameter ratio greater than 1,000,000. After their discovery, CNTs have drawn extensive research attention due to their excellent material properties. Their physical, chemical and electronic properties are excellent and their composites provide great possibilities for enormous nanometer applications. The current study provides a systematic review based on prior literature review and data gathered from various sources. The various research studies from many research labs and organizations were systematically retrieved, collected, compiled and written. The entire collection and compilation of this review concluded the use of CNT approaches and their efficacy and safety for the treatment of various diseases such as brain tumors or cancer via nanotechnology-based drug delivery, phototherapy, gene therapy, antiviral therapy, antifungal therapy, antibacterial therapy and other biomedical applications. The current review covers diverse applications of CNTs in designing a range of targeted drug delivery systems and application for various therapies. It concludes with a discussion on how CNTs based medicines can expand in the future.

## 1. Introduction

In recent years, nanotechnology is considered as a major technology for improving the quality of life and for promoting the health of society; accordingly, there is an increase in financial support by different government agencies for various research studies on nanomaterials [1]. Nanotechnology involves the study of manipulation of matter on an atomic and molecular scale. Based on in vitro as well as in vivo studies, different novel formulations have been developed in order to enhance therapeutic efficacy and also to decrease the undesired effects produced [2]. Various nano sized drug delivery carriers have also been evaluated in recent years, and one such carrier is the nanotube (a synthetic material of carbon atoms) [3]. Nanotubes come in many forms, viz. short, long, open, closed, single walled, double walled, multi walled or in different types of spiral structures. Nanotubes are constructed according to their length-to-diameter ratio, which is up to 132,000,000:1, and are considerably greater when compared to other materials. These cylindrical hollow carbon molecules have novel properties that have a myriad of applications in optics, electronics, nanotechnology and other fields of material science [4]. Carbon nanotubes (CNTs) are one-dimensional carbon allotropes with a nanostructure that can have a length-to-diameter ratio greater than one million and are formed by rolling a thick sheet of graphene into a smooth cylinder with a diameter of the order of a nanometer (nm). They also have high mechanical strength, rich electronic properties, large surface area, light weight, chemical stability and excellent thermal stability [5]. Sumio Iijima from Japan was the first scientist who discovered CNTs in 1991. Due to their tubular structure, these carbon carriers have different novel approaches that make them useful in diverse applications such as mechanical electronic, biomedical, structural, thermal, optical and various other fields of medicinal science and engineering. CNTs show astonishing strength and also have unique electrical properties.

CNTs are considered as building blocks of nanotechnology. They are chosen widely for their high loading of cargo molecules and excellent cell penetration ability, which enables them to deliver drug substances into neoplastic cells for selective destruction and to reduce the distribution of drugs to normal adjacent cells that aids in avoiding the toxicity of the cells, mainly in cancerous cells [6]. CNTs are poorly soluble in both mediums (aqueous and organic) as they tend to agglomerate in the medium. This was a major problem in biomedical and biological applications; hence, to overcome this, functionalization was made recently by modifying the chemical structure of CNTs to disperse and to solubilize them in a medium [7,8,9,10,11]. Covalent modification is the modification of CNTs performed by direct sidewall functionalization that results in high solubility, high structural alterations, degradation capacity, high drug loading capacity and low toxicity. On the other hand, non-covalent modifications are performed by wrapping biopolymers, surfactants and polymers that result in solubility at high levels, high structural alterations, degradation capacity to a moderate level, high drug loading capacity and low toxicity.

CNTs release the drug in a controlled manner, and they are site specific. They load the high amount of drugs owing to their hollow tubular structure, and they can also increase the efficiency of therapeutic molecules. CNTs deliver therapeutic drugs as well as moieties into the cancerous cells via endocytosis or by penetration into the cell without causing apparent cell damage. As a result, CNTs can be used in cancer therapy as drug delivery carriers for the targeted drug delivery and also in various therapies. They have other biomedical applications too, thereby indicating an example of a true nanotechnology. Therefore, this review covers the various diverse applications of CNTs in various targeted drug delivery systems and various drug therapies. It concludes with a discussion on how CNT medicine can expand in the future.

## 2. Types of CNTs

The term nanotube usually refers to the carbon nanotube (CNT), which has received enormous global attention from researchers of various fields over the last few years. CNTs are classified into three types: single-walled CNTs (SWCNTs), double-walled CNTs (DWCNTs) and multi-walled CNTs (MWCNTs). SWCNTs consist of a single layer graphite sheet that is enfolded in the form of cylindrical tube, whereas MWCNTs comprise an arrangement of SWCNTs concentrically placed one inside another, which resembles the rings of a tree trunk.

### 2.1. SWCNTs

SWCNTs are an important part of the nanotube world and consists of a single graphene layer that is embedded within the cylindrical hexagonal structure by Van der Waals forces, thereby making them effortlessly twistable and more flexible [12,13]. SWCNTs are novel drug delivery carriers known for the transporting drug molecules, proteins and nucleic acids. Multifunctional SWCNTs can improve drug therapeutic effectiveness by minimizing its toxicity [14]. Solubility of SWCNTs can be investigated by suspending them in Polysorbate-80, which can be used to investigate the solubility and toxicity [15]. SWCNTs can penetrate into the cell membrane more easily with increased loading capacity of drugs and prolonged circulation of half-lives. These properties have made SWCNT a promising nanocarrier for chemotherapy and cancer diagnosis. SWCNTs are harder to make when compared with MWCNTs and exhibit electric properties that cannot be shared by MWCNTs variants [16]. SWCNTs are the suitable carriers for delivering drug molecules into tumor cells when compared with MWCNTs, [17] and as they have the ability to penetrate readily into the cells, SWCNTs have proved beneficial in cancer therapy, bone cell growth and imaging. This advantage of SWCNTs demonstrates that they are potential nanocarriers for safe and effective transport of drugs [18].

### 2.2. DWCNTs

DWCNTs are also a type of CNTs that are structurally similar to SWCNTs, but the difference is that DWCNTs consist of two concentric layers containing a cylindrical tube inside an outer layer of it. CNTs based on the chirality are classified as armchair, zigzag and chiral. Armchair is an arrangement of bonds where the vectors are perpendicular to the tube axis. Zigzag is an arrangement where the V-shaped tubes are perpendicular to the axis. Finally, chirals are different from armchair and zigzag types, and other than the above properties, they are also classified on the basis of structural transformation, preparation technique, solubility characters and many more.

### 2.3. MWCNTs

MWCNTs are made from SWCNTs that generally appear similarly to Russian dolls and have a concentric cylindrical graphite tube made up of graphite layers that are rolled multiply; thus, it has a large surface area and offers increased drug loading capacity [19]. Large quantities of MWCNTs can be produced easily at a reasonable price. The main techniques used in the development of MWCNTs include electrical arc and chemical vapor deposition. Due to sp^2^ hybridization in MWCNTs, a delocalized electron is formed along the wall, which causes π–π interactions between the adjacent cylindrical layers of MWCNTs, thereby ensuing structural defects and less flexibility [20,21,22].

## 3. Synthesis of CNTs

**Figure 1:**
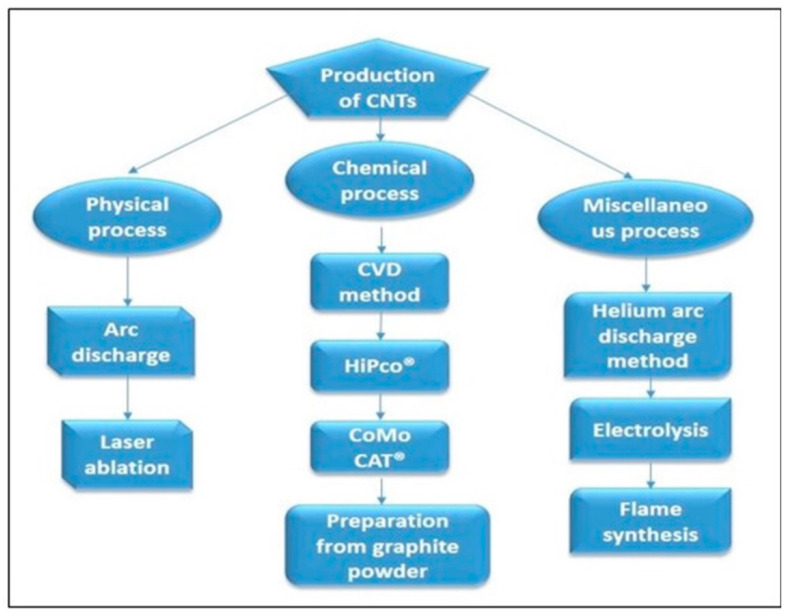
Production processes for carbon nanotubes (CNTs).

Various techniques are available for synthesizing CNTs. Generally, to synthesize CNTs, any one of the three major processes can be used, such as chemical processes, physical processes and miscellaneous processes (Figure 1). Moreover, many developments in catalysis and growth processes rendered these CNTs commercially viable.

### 3.1. Physical Processes

By using the physical principles of carbon, the most common methods for the conversion of carbon into CNTs include the arc discharge method (ADM) [23,24] and laser ablation method (LAM) [25]. Arc discharge process in CNT synthesis uses high temperatures (above 1700 °C) often resulting in an increase in CNTs with less structural defects compared to other processes. Arc discharging occurs between high-purity (6–10 mm optical density) graphite electrodes and normally water-cooled electrodes between 6 and 12 mm in diameter and divides them into 1 to 2 mm into a helium filled room (500 torr) at atmospheric pressure (hydrogen or methane atmosphere can replace helium). The chamber includes the graphite anode, cathode, evaporated carbon molecules and a variety of metal catalytic particles of metal (e.g., nickel, iron and/or cobalt). Direct current is transmitted through an arcing process (camber), and the cabinet is heated to approximately 4000 k. During the arcing process, half of the evaporated carbon hardened with the tip of the cathode with 1 mm/min of deposit forming so-called “cylindrical hard deposits or cigar-like structure,” and an anode is consumed. The remaining carbon (solid gray shell) is attached to the edges and condenses to the “chamber soot” near the chamber walls and the “cathode soot” to the cathode. The inner core, the chamber soot and the cathode soot are soft and dark, which produce either SWCNTs or MWCNTs and polyhedral graphene particles.

The analysis of cathode deposits in which soft and dark inner core deposits consist bundle-like structures containing nanotubes arranged randomly with a gray external shell, which consist of curved and solid graph layers, is evaluated by scanning electron microscopy (SEM). There are two key methods in arc discharge deposition and CNTs synthesis, such as synthesis by using different catalyst precursors (for SWCNTs) and synthesis without catalyst precursors (for MWCNTs). The synthesis of SWCNTs utilizes different catalyst precursors and a complex anode made up of metal and graphite (e.g., Fe, Co, Pd, Pt, Gd, Ag and Ni or mixtures of Co, Ni and Fe with other elements, e.g., Co-Ru, Co-Pt, Fe-Ni, Ni-Y, Co-Cu, Ni-Cu, Co-Ni, Ni-Y, Ni-Ti, Fe-No, etc.). Without the use of catalyst precursors, the synthesis of MWCNTs can be achieved. The key benefit of the arc-discharge technique is its ability and efficiency to yield a large quantity of nanotubes; consequently, the key drawback of that method is comparatively little control over the alignment including the chirality of synthesized nanotubes, which is essential for their characterization and application. Moreover, purification of the final product is essential, as the metallic catalyst is required for the reaction.

LAM involves “high-power laser vaporization, a quartz tube containing a block of pure graphite heated inside a furnace at 1200 °C in an Ar atmosphere, in which the object of using laser is vaporizing the graphite within the quartz.” As the synthesis of SWCNTs by the process of arc-discharge, the laser technique includes the addition of metal particles as catalysts to graphite targets in order to produce SWCNTs. As per the studies, the diameter of the CNTs depends on laser intensity; thus, the diameter of the tubes becomes narrower as the laser pulse power increases.

The concepts and principles of the LAM are identical to ADM, but in case of LAM, a laser strikes a pure graphite pellet that contains catalyst materials such as nickel or cobalt, providing the required energy. The key benefits of laser ablation include significantly higher yields and low metallic impurities. The metallic atoms involved have a tendency to evaporate from the end of the tube once it is closed. However, the drawback is that the nanotubes obtained are not inherently straight and have certain branching. Unfortunately, LAM is not economical since the procedure includes high-purity graphite rods and great levels of laser powers (two laser beams in some cases); moreover, the quantity of nanotubes synthesized regularly is not as high as ADM.

### 3.2. Chemical Processes

**Figure 2:**
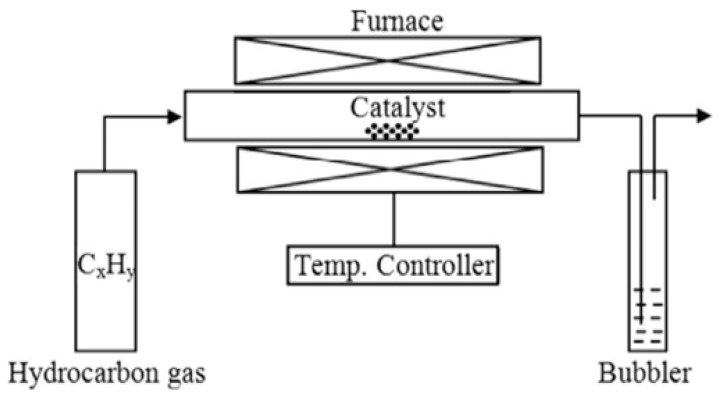
Schematic presentation of a CVD setup (image reproduced from reference [27], which was published under a creative common attribution (CC BY) license).

The chemical processes involve the synthesis of CNTs from graphite powder by treatment with acids such as nitric acid and sulfuric acid, and precipitation can be performed with potassium chlorate or other precipitating agents. Various methods are used in the chemical process, such as the chemical vapors deposition (CVD) method [26,27], high pressure carbon monoxide reaction (HiPco^®^) [28] and the cobalt and molybdenum (CoMo) CAT^®^ process [29]. Chemical vapor deposition (CVD) is one of the standard methods for producing CNTs that enables CNTs to proliferate into various materials and includes the chemical breakdown of a hydrocarbon on a substrate. A schematic presentation of a CVD setup is depicted in Figure 2 [27]. The main process for the growth of CNTs by this method is to excite carbon atoms with metallic catalyst particles; the layer of metal catalyst particles (cobalt, nickel, iron or blend) prepares and processes a substrate at approximately 700 °C. The first step is the expansion of nanotubes in which two kinds of gases, including as gasses containing carbon such as acetylene, ethylene, methane or ethanol and process gas such as nitrogen, hydrogen or ammonia, fuel the reactor. Carbon-containing gas breaks at the surface of catalyst particles; thus, carbon is visible at the edges of the nanoparticles where nanotubes can develop. This process is still under debate, and the generally accepted models include tip growth. During growth and expansion, catalyst particles are able to sustain their adhesion or bond between the substrate surface and catalyst particles remains at the nanotube base or the nanotubes. In comparison to LAM, CVD is an economical method for large-scale production of CNTs with better purity; hence, the key benefit of CVD includes high purity materials obtained with simple reaction control.

### 3.3. Miscellaneous Processes

These are the less used processes or methods for the synthesis of CNTs, which include the helium arc discharge method, electrolysis [30] and flame synthesis [31]. George Washington University researchers found a new pathway in 2015 for synthesizing MWCNTs via electrolysis of molten carbonates, and the mechanism is similar to that of CVD. Some of the metal ions were condensed to metal form followed by an attachment on the cathode as a core point for CNTs growth.

## 4. Characterization of CNTs

**Figure 3:**
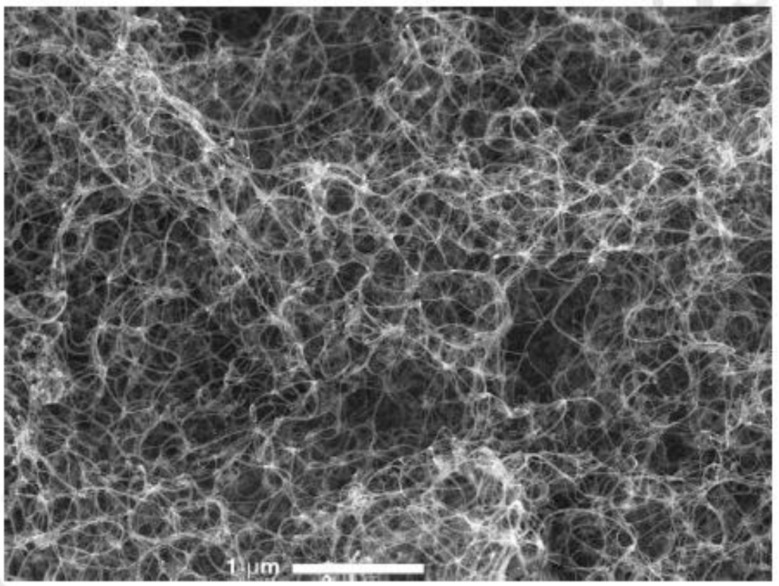
Scanning electron micrograph of CNTs (image reproduced with permission from reference) [23].

**Figure 4:**
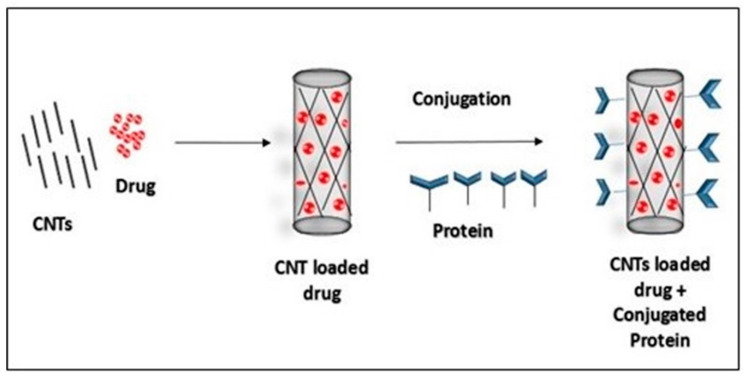
Loading of drug into CNTs and protein conjugation of CNTs.

Various methods are used to characterize CNTs, including particle size distribution, zeta potential [32], scanning electron microscopy (SEM), scanning tunneling microscopy (STM), X-ray photoelectron spectroscopy (XPS) [33], X-ray diffraction (XRD), transmission electron microscopy (TEM) [34,35], neutron diffraction, infrared spectroscopy (IR) [36] and photoluminescence spectroscopy. These characterization techniques can be employed for characterizing native as well as drug loaded CNTs. SEM can determine the surface morphology of CNTs. The samples were tested in various magnifications at appropriate accelerating voltages. Software for image analysis (Amrita University, Cochin, India) was used to obtain both an automated shape and surface morphology analysis. The weighted quantity of the sample was attached to a double-sided aluminum plate. The sample was then covered with a fine skinny layer of gold in an aluminum plate in order to make the sample conductive and then subjected to SEM analysis; the image was captured and recorded. SEM analysis can be used for analyzing the tubular structure of CNTs (Figure 3). For determining the loading efficiency, a required volume of CNTs, loaded with a drug (Figure 4), is diluted with a known volume of suitable solvent and then sonicated. It is then subjected to centrifugation in order to remove excess CNTs from the formulation. The supernatant solution is then collected and used in order to determine the concentration of drugs by using UV-visible spectrophotometry.

## 5. Targeting and Uptake of CNTs

### 5.1. Cellular Uptake of CNTs

**Figure 5:**
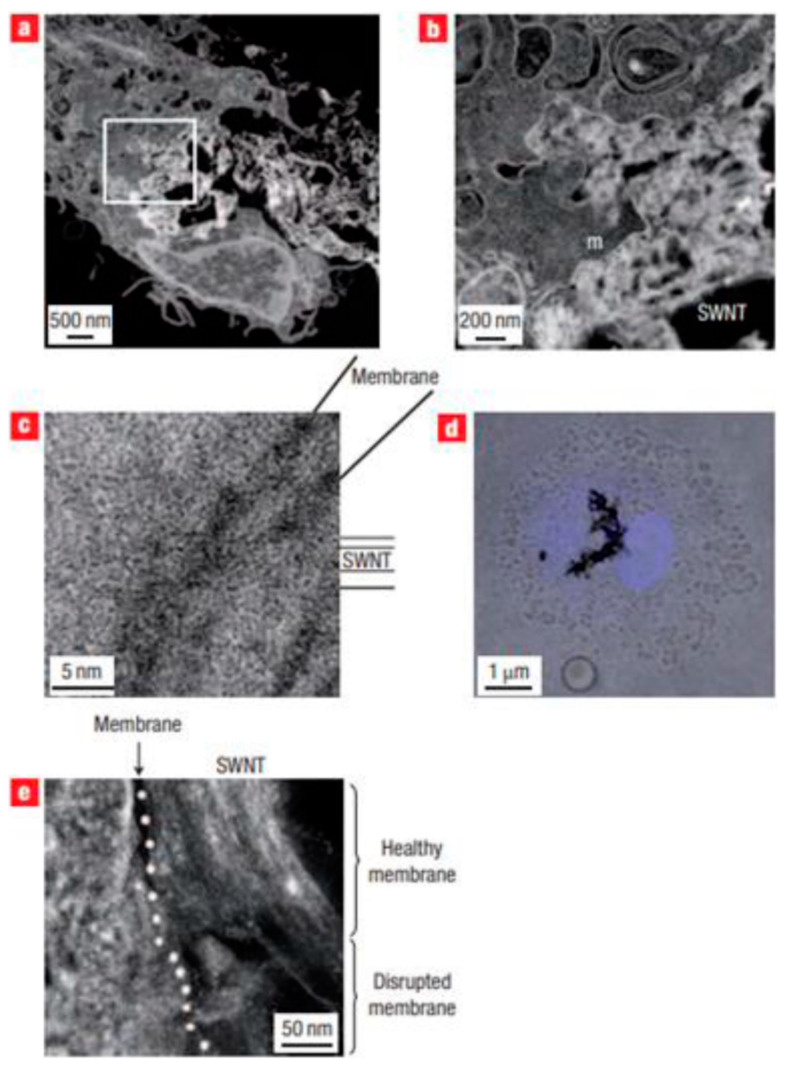
Localization of intracellular SWNTs within stained cell sections. (**a**) HAADF-STEM image showing SWNT bundles being actively ingested by a phagosome at 4 days. (**b**) Higher magnification image of boxed area in image a illustrating that SWNTs were compartmentalized inside the phagosomal membrane (m). (**c**) Bright-field STEM image of SWNTs translocating across the lipid bilayer into the neighbouring cytoplasm. (**d**) Confocal microscope image of HMM exposed to AgI@SWNT at 3 days, confirming inclusion of SWNT bundles inside the nucleus (blue). (**e**) HAADF-STEM image showing SWNTs within a lysosome, with membrane disruption at the region where SWNTs fused with the membrane (image reproduced with permission from reference) [39]. Copyright 2007 Springer Nature.

**Figure 6:**
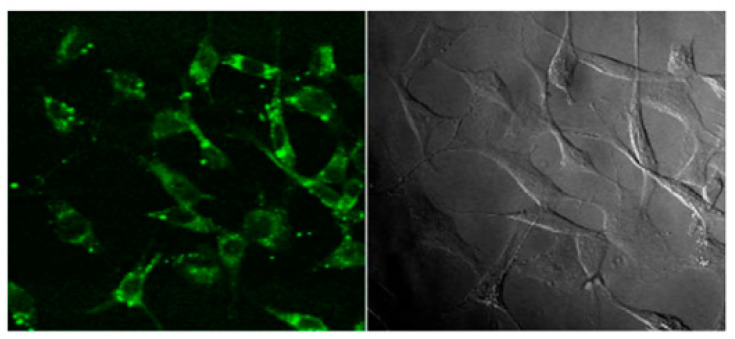
Confocal microscopy images of 3T6 cells post incubation with fluorescent CNT (image reproduced with permission from reference) [41].

Various studies have confirmed the uptake of CNTs into cells; however, the mechanism involved in the penetration of CNTs into the cells is still not well understood [37]. Tagging the CNTs with different types fluorescent materials such as dye-based quantum dots allowed researchers to track the exact uptake of CNTs [38]. Moreover, the cell uptake of CNTs can also be tracked by applying advanced microscopic techniques such as transmission electron microscopy (TEM) or atomic force microscopy (AFM) analysis; one such example is presented in Figure 5 [39]. CNTs, due to their needle-like shape, are able to penetrate into the various components of the cell without causing any apparent cell damage [40,41]. Perpendicular positioning of the CNTs relative to plasma membranes shows that uptake of CNTs is similar to that of nanoneedles, which diffuse through the plasma membrane without causing necrobiosis. In order to investigate the uptake of CNTs into cells, fluorescent proteins can be attached, and they can be identified within endosomes. The cellular uptake of CNTs occurring via endosomes can then be estimated [42]. The uptake mechanism depends on the length of CNTs [43], and those that are shorter than 1 μm are extremely simple to interiorize into cells. With the utilization of epifluorescence and confocal microscopy, the functionalized CNTs labeled with a fluorescent agent show that CNTs will penetrate into the cytoplasm through the cell or through the nucleus of fibroblasts. Figure 6 represents confocal microscopy images of 3T6 cells post incubation with fluorescent CNT depicting CNTs internalization [41]. An in vitro CNT nanoinjector system was developed [44]. By using atomic force microscope tip and by functionalizing the MWCNTs attached to a cargo compound via a disulfide linker, a nanoinjector was designed and developed. This might be easily transported into the cell wall, where the disulfide bond will break inside the cell, thereby resulting in the discharge of the cargo compound inside the cytosol. A cytotoxicity study of SWCNT complexes utilizing platinum suggests that it can deliver platinum to improve the cellular uptake of the drug [45].

### 5.2. Cell Viability and Proliferation Assay

In order to assess cell viability and proliferation, stem cells were cultured with fetal bovine serum and streptomycin or penicillin in Dulbecco’s Modified Eagle Medium. It was then maintained at room temperature in a carbon dioxide incubator for a few days, and CNTs with various concentrations are then added into the culture medium. Stem cells without CNTs are treated as control, and the media are exchanged once in three days by carrying out an assay in triplicate [46].

### 5.3. Trypan Blue Assay

Trypan blue is a vital dye, and its reactivity depends on the mechanism where the negatively charged chromophore reacts with the damaged cell membrane and interacts with the cell. As a result, all cells that exclude trypan blue are viable. In addition, cells are released with trypsin-ethylenediaminetetraacetic acid (EDTA) and are washed and suspended in a test tube. A specific amount of the cell is then taken and transferred into another Eppendorf tube. Trypan blue dye is then added and mixed. A cell suspension containing trypan blue is drawn and filled in the hemocytometer. The dead cells are stained blue and are counted separately for the viability count.

### 5.4. 3-(4,5-Dimethylthiazol-2-yl)-2,5-Diphenyl Tetrazolium Bromide (MTT) Assay

MTT assay is conducted to assess stem cell viability. The stem cells are treated with increasing concentration of CNTs that are prepared in a proper medium, and the medium of each well plate is removed. The plate is then washed with phosphate buffer solution which is again replaced with serum-free medium and MTT solution; it is then incubated at 37 °C for four hours in a carbon dioxide humidified incubator. This results in the formation of purple formazan crystals in the plate. Then, the medium is removed, and dimethylsulfoxide is added to each well plate. The plates are then incubated in a dark location. A purple color forms since DMSO dissolves in the formazan crystals. This is then transferred into a well plate, and the absorbance is recorded in an ELISA reader. Three different experiments are generally repeated independently for minimizing errors [47,48].

### 5.5. Flow Cytometry

Cell surface markers of stem cells, which are treated with CNTs, are evaluated by flow cytometry. After treatment with trypsin-EDTA, the cells obtained are centrifuged, and the cell plates are washed with phosphate buffer solution. Cell plates are then incubated with an antibody against conjugated phycoerythrin/fluoroisothiocyanate in a dark place at 4 °C. Non-specific fluorescein isothiocyanate (FITC) conjugated with IgG can be substituted for the primary antibodies in an isotype-controlled system, and flow cytometry is performed to determine cell surface markers [49].

## 6. Toxicity of CNTs

Owing to their unique mechanical, electrical, chemical and physical properties/characteristics, CNTs have gained a great deal of attention from researchers and have proven its potency and applicability across an array of disciplines. Considering the demand as the CNTs are been produced at large-scale, their exposure to the general population has increased dramatically either indirectly or directly. This has prompted safety concerns related to the effects of CNTs on human health and issues related to their toxicities. Although there has been rigorous research performed in this direction and considerable experimental reports and data allied to CNTs toxicity at the cellular, molecular and animal levels are available, the conclusions do not align and often conflict. Thus, systematic CNT toxicity understanding that is more inline is the most needed piece of the puzzle.

Cell culture toxicity studies of pristine nanotubes have reflected CNTs to be toxic to cells due to the oxidative stress induced by SWCNTs [49,50,51]. Functionalized and highly water soluble pristine nanotubes are less cytotoxic than compared to non-functionalized pristine nanotubes [52,53]. The in vitro cytotoxicity assays of CNTs include MTT assay, neutral red uptake assay and lactate dehydrogenase release assay to list a few. Morphological changes of CNTs and flow cytometry analysis have also proven their efficiency in marking the characteristics of cytotoxicity. The toxicity of CNTs is additionally associated with their lengths. In the case of drug delivery within the field of medicine, CNTs are observed as better potential carriers. However, the toxicity that results is not understood properly and holds high potential for exploration. Normally, the toxic effects of nanoparticles arise from numerous factors; out of that, high surface area and intrinsic toxicity of the surface are the most affecting crucial factors. Nanoparticles under one hundred nanometers are considered to possess enough potential to be more toxic to the respiratory system and might spread from the site of deposition and escape from regular phagocytic defenses. In addition, it can alter the structure of proteins that activate inflammatory and immunologic responses, which can have an effect on the normal tissue function [54]. To determine the toxic effects of CNTs, in vitro and in vivo analyses were performed. The toxic effects of CNTs are generally produced due to the presence of ferric impurities and the length of CNTs [55]. Some other parameters resulting in the toxic effects of CNTs include physical form, structure, surface charge, surface chemistry, agglomeration state and the degree of functionalization as well as the purity of the samples being studied [56]. Moreover, the toxicity of CNTs depends on the degree of functionalization and the presence of functional groups. A synthesis of many batches of non-functionalized pristine CNTs has been shown to contain impurities such as amorphous carbon and metallic nanoparticles, acting as another source to produce toxic effects. Various studies have suggested that SWCNTs are able to induce cellular responses by activating molecular signaling associated with oxidative stress [57]. When CNTs are administered through various routes in mice; they showed many adverse effects including the accumulation of CNTs in major organs such as spleen, liver and lungs. Intravenous exposure of CNTs in increasing concentration has shown negligible amounts of toxic effects [58]. After intravenous administration of SWCNTs coated with polyethylene glycol (PEG), they were removed slowly from the body with no toxicity detected during the excretory process [59].

In another research attempt, Ema et al. have aimed at evaluating the effects of SWCNT’s length on pulmonary toxicity in the rat model [60]. Each subject has undergone intratracheal instillation with a short (around 0.40 μm) and long (around 2.77 μm) SWCNT and was observed for 6 months. Neither of the instilled SWCNTs has affected body weight, autopsy findings or clinical signs during the study period. On the other hand, a noticeable difference was noted in the composition of bronchoalveolar lavage fluid (BALF) amid the treated subjects of both groups. The short SWCNTs were reported to cause higher inflammation and persistent lung injury with respect to long SWCNTs. Although changes in lung histopathological features and BALF parameters were noted in both treated groups, the severity of change was elevated in the case of short SWCNT subjects. Thus, the researchers concluded that the length of SWCNTs critically affects the severity of pulmonary toxicity post intratracheal instillation [60].

In another similar sort of in vivo toxicity research vocation, Fujita et al. have fabricated two types of SWCNTs, viz., short-linear shape thin bundles (CNT-I) and long-linear shape thick bundles (CNT-II) and instilled these via the intratracheal route in rats [61]. Additionally, they have also performed cell-based in-vitro assays (using NR8383 rat alveolar macrophages). MIP-1α expression, levels of total protein, histopathological outcomes and BALF cell counts had depicted that CNT-I resulted in a high degree of pulmonary inflammation with sluggish recovery when compared with CNT-II. Furthermore, comprehensive gene expression analysis outcomes reflected that cell proliferation and immune-inflammatory responses in the subjects were strongly associated with CNT-I-induced genes post 7th and/or 30th day of instillation. On the other hand, downregulation or upregulation of several genes was experienced by the CNT-II group after the first day of instillation. Phagocytosis of both CNT-II and CNT-I SWCNTs by NR8383 cells was observed. It has been noted that treatment with CNT-II resulted in MIP-1α expression, ROS production, cell growth inhibition and multiple genes were involved in stimulus response, whereas treatment with CNT-I had not exerted any significant response in these fronts. All these outcomes suggested delayed pulmonary inflammation with very slow recovery in the case of short-linear shaped thin bundle SWCNTs. On contrary, long-linear shaped thick bundles have delicately resulted in alveolar macrophages cellular responses and depicted acute inflammation post inhalation. These findings establish the fact that SWCNT-allied pulmonary toxicity relies significantly on the bundles’ size, and the aforementioned physical parameters are of great significance in assessing and managing toxicity risks allied with SWCNTs [61].

Moreover, after subcutaneous administration of CNTs, some reactions such as toxicity, drug allergy or ulceration were observed, but no adverse reaction and no agglomerates were deposited in lungs, spleen and liver. Many researchers have shown that only a lesser amount of CNTs can enter into the blood stream, thereby avoiding toxicity by altering the route of administration. In one of the studies, it is stated that many cellular pathways that can cause DNA damage can be triggered by CNTs [62]. Therefore, thorough investigations focusing on and exploring cellular pathways are needed in order to investigate the mode and extent of DNA damage.

## 7. Strategies to Overcome CNTs Toxicity

The toxicity issues of SWCNTs and MWCNTs are chiefly reliant on their physicochemical characteristics such as aspect ratio, size, chemical composition, shape, crystal structure, stability, surface area, surface roughness, surface energy and surface charge [63,64]. Thus, for reducing the CNTs allied toxicities, modulating and/or altering these characteristics would serve as an ultimate approach. Considering the wide range of applicability of CNTs based systems in the biomedical and healthcare sectors, there is an enormous need for guidelines for designing safer CNTs.

The surface charge is a very crucial factor that affects the interaction of CNTs with biological systems; hence, one can alter or modulate the surface charge of CNTs in order to reduce allied toxic manifestations [65,66]. To date, researchers have applied diverse methods for attaining this particular surface characteristic modulation. One research group led by Gilbertson et al. has developed a novel statistical model relating the surface charge of CNTs with zebrafish mortality at their embryonic stage [67]. The zebrafish models are the preferred choice for in vivo evaluations in the vertebrate as they have physiological and molecular conservations with the rest of the vertebrates during their embryonic developmental stage [68]. The researchers closely monitored zebrafish bioactivity post-exposure relative to fully characterized and systematically modified MWCNTs. The four physicochemical characteristics under consideration were electrochemical activity, percentage of surface oxygen, dispersed aggregate morphology and size and surface charge. Analysis outcomes have established surface charge as the best predicting factor of zebrafish mortality post 24 h of fertilization. Multiple conclusions were drawn by applying multivariate statistical methods based on a model that zeroed in on physicochemical characteristics that can superlatively estimate the probability of adverse response occurrence. Thus, defining and correlating this property-risk relationship has established a basis for further expansions aimed at designing novel low toxicity CNTs.

Apart from the modulation of physico-chemical characteristics, detailed materials, structural characterization, mode and extent of uptake by the exposed cells and CNTs elimination from the administered body compartment along with the elimination mechanisms need to be deeply explored. Moreover, there is a need to fuel research in order to obtain more details about toxicity mechanisms and to develop new test systems that would systematically evaluate and provide indications of the nanotoxic nature of newly developed CNTs for risk assessment.

## 8. Applications of CNTs

### 8.1. CNTs in Cancer Targeted Drug Delivery

Present day anticancer treatments, such as removing cancerous cells (surgery), radiation therapy or killing cancerous cells (chemotherapy) using anticancer drugs, causes damage to healthy cells as well [69,70]. CNTs due to their unique features, such as cellular uptake, high drug loading and thermal ablation, are used as a carrier for delivering drugs into tumor cells. Due to hydrophobic nature of CNTs, they are insoluble in water, which results in limitations with respect to their use in biomedical and medical chemistry applications. Hence, CNTs are functionalized to be used as a drug delivery or carrier system. Different functionalization methods are available such as adsorption, covalent bonding and electrostatic interaction for reducing the hydrophobic nature of CNTs and to help evade the aggregation of CNTs, thereby facilitating their use in various applications [71]. Tumor cells are fast growing, uncontrolled and proliferative with high metabolic rates, which increases the demand of oxygen and nutrients; this results in the formation of new vessels for the supply of oxygen and nutrients. Tumor cells obtain energy through the glycolysis pathway and release growth factors and enzymes, thereby resulting in imbalance in angiogenic regulators that dilate the blood vessels of cancer cells. Consequently, this results in large gap junctions between endothelial cells [72]. Due to enhanced vascular permeability and inadequate lymphatic drainage at the cancer cells, it enables passive targeting by using various polymers in the form of nano vectors, which depend on surface charge, molecular weight and polymer nature [73,74]. Moreover, due to a larger inner volume of CNTs, it permits the encapsulation for each low and high molecule of drugs. This encapsulation protects the loaded drug from the outside environment, which allows passage through the polymeric membranes. Additional drugs may also be loaded into the CNTs in the case of dual-drug therapy. The site of the drug to be delivered by the CNTs could be internal or external. Internalization or encapsulation depends on the van der Waals forces, and they are used for the drugs that are unit sensitive to external environments and that can be easily broken down [75].

CNTs-based carrier system offers the oral administration of erythropoietin, which has not been used as a result of the denaturation of erythropoietin by the stomach, surrounding conditions and enzymes. In the case of a drug delivery system based on CNTs, attachment of the drugs to appropriate carriers will increase their bioavailability by increasing residence time within blood circulation and by enhancing solubility. The efficacy of the drug may be increased by CNTs by targeting a particular site and accumulation within the pathological zone known as the therapeutic-effects-related site. CNTs have a novel capability to penetrate into cell membranes, and this provides the simplest method to deliver drug molecules into the cytoplasm and nucleus. Different intrinsic properties of CNTs such as Raman and photoluminescence might provide several extra functions to the CNTs for the chase [76]. The major question is how drug-loaded CNTs recognize and enter into a particular target site for a desired action of the anticancer drug. Various methods are available for investigating how neoplastic drugs loaded into the CNTs recognize the target site, and one strategy is coating the surface of the CNT with a selected protein having an affinity for the target neoplastic cell [77].

**Figure 7:**
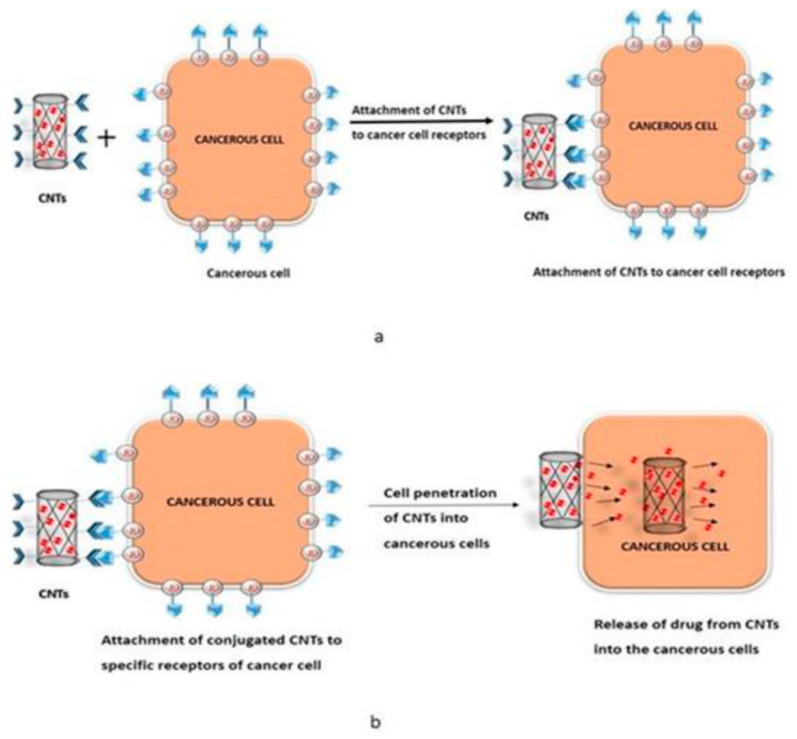
(**a**) Attachment of CNTs to cancer cell receptors. (**b**) Release of drug from CNTs into cancerous cells.

Carbon nanohorns are the sphere-shaped masses of CNTs possessing an irregular shape that act as a possible carrier for the drug delivery system. SWCNT and MWCNT forms are extensively used for the delivery of drug molecules and biomolecules into the tumor cells [78]. Conjugation of the drug molecules with suitable conjugating agents along with the coating of polymers avoids the random bioavailability of relatively low molecular weight drugs and enhances site specific targeting of drug molecules. Various studies have shown that cancer cells overexpress folic acid receptors (Figure 7a,b).

Researchers from various fields have concluded that the nanocarriers such as CNTs can be surface engineered to which folic acid can be attached in order to improve site-related responses for cancer targeting. In a research attempt, a novel active targeted-cum-pH responsive system has been developed for DOX delivery to cancerous cells, implying folic acid (FA) conjugated MWCNTs. Therein, first the covalent conjugation of acid treated MWCNTs was performed with polyethyleneimine (PEI), which then has sequentially been modified with FA, fluorescein isothiocyanate, triethylamine and acetic anhydride to obtain FA-bound multifunctional MWCNTs. The resultant nanocarriers exhibited superior biocompatibility and excellent colloidal stability. Moreover, FA bound MWCNTs depicted pH-responsive DOX release in acidic environments and comparatively higher DOX loading up to around 70.4%. Furthermore, the in vivo experiment outcomes have established that fabricated CNT-based novel nanocarriers have not only effectively suppressed tumour growth but also reduced free DOX allied side effects. Thus, the researchers have proposed clinical translation of their developed FA-conjugated MWCNTs [79].

In yet another research vocation, Jawahar et al. have developed CNTs loaded with raloxifene hydrochloride (RLX), implying a modified Staudenmaier process. A quality-by-design approach was undertaken, and they noted that stirring speed and temperature were the most prominent factors influencing entrapment and the particle size of RLX loaded CNTs. Surface functionalization of the prepared CNTs was performed using FA with the intent of delivering RLX only to breast cancerous cells in a target-oriented mode. Outcomes of in vitro drug-release studies confirmed a pH responsive RLX release from the system, as it was anticipated. Furthermore, in vitro cytotoxicity study data evidently depicted FA conjugated CNTs efficacy with a higher degree of affectivity and promising apoptosis induction against cancer cell lines (IC50 value reported was around 43.5 μg/mL) [80].

Another group of researchers opted for a bifunctional nanoplatform to design and develop a chemo-photothermal approach-based targeted and synergistic cancer therapy. Using facile methodologies, they first constructed a nanoplatform by coating poly(N-vinyl pyrrole) onto cut MWCNTs, which were then linked with FA-polyethylene glycol via a thiol-ene click reaction in order to achieve water dispersibility, higher circulation time in blood, biocompatibility and precise targeting. The poly (N-vinyl pyrrole) coating on MWCNTs not only resulted in enhanced photothermal efficacy but also offered multiple sites for tailoring the drugs and targeting molecules on CNTs. The developed biofunctional CNTs nanocarriers have depicted higher loading capacities and augmented pH responsive unloading for model anticancer drug DOX and many other anticancer agents. Hence, due to their exceptional photothermal conversion efficiency and drug targeting capability, the researchers had proposed utilizing these biofunctional nanocarriers as chemo-photothermal-cum-chemotherapeutic nano agents for augmenting cancer therapy [81].

CNTs are able to deliver therapeutic drugs and moieties into cancerous cells by endocytosis and other mechanisms [82,83]. SWCNTs and MWCNTs, due to their high retaining capacity to accumulate in lymph nodes, prolong the duration of action when compared with other nanocarriers. Hence, many investigators have shown that conjugation or functionalization of low molecular weight drugs along with the suitable polymers avoids random bioavailability and enhances target specificity [84].

Cisplatin, an antitumor category drug, was developed into magnetic nanoparticles, and they were loaded into MWCNTs and functionalized with B complex. With the impact of an external magnet, CNTs are targeted into lymph nodes, and they release the drug at a slower rate in order to prolong the duration of action of the drug and to inhibit the expansion of tumor cells. The ideal properties of carbon tubes for showing its effects on cancer cells include the following: they should have its own targets, they should have sufficient adsorptive effects on the cancerous cell for the transportation of cells and they should have the capability to release the drug at its relevant site.

In a recent study, an anticancer drug, gemcitabine, was loaded into magnetic MWCNTs and injected subcutaneously on the mice model. It demonstrated high activity against lymph node metastasis [85]. Various approaches were applied in order to load the drug molecules into the sidewalls of functionalized CNTs and graphene-based nanomaterials by covalent or non-covalent attachment [86,87]. 

Polyampholyte-grafted-SWCNTs were prepared by a green process in order to enhance cancer therapy. N-isopropyl acrylamide CNT enhances the activity of doxorubicin in cancer therapy. Ethylenediamine functionalized with the SWCNTs was used to suppress p53 gene relative to breast cancer MCF-7cells. Nanotubes consisting of triple beta D glucan have increased the activity of selenium particles for their anti-luekemial properties. Bioadhesive polymers such as chitosan and sodium alginate are used to enhance the water solubility of the CNTs, and folic acid can be used for improving the targeting properties of CNTs used in cancer therapy [88].

### 8.2. CNTs in Brain Targeted Drug Delivery

Acetylcholine deficiency potentially results in the development of Alzheimer’s disease and results in memory loss and cognition. Acetylcholine is hydrophilic in nature, and it is difficult for it to cross the hydrophobic blood–brain barrier (BBB). Acetylcholine non-covalently loaded into SWCNTs was the novel strategy used to increase the penetration of drugs into BBB [89]. The in vitro and in vivo studies on functionalized CNTs have shown promising results with improved carrier biocompatibility and targeting [90]. Carboxylated SWCNT can be used as sustained release nanocarriers for delivering levodopa with respect to Parkinson’s disease, and results have revealed that the synthesized nanohybrid did not compromise the viability of cells. These can act as efficient carriers of drugs [91]. Cell penetrating peptide and cancer-targeted molecule-MWCNT can be used as new effective strategies as next-generation nanodrugs for penetrating the BBB for precise orthotopic glioma therapy. The trans-activating transcriptional activator (TAT) can enhance the permeability of brain endothelial cells by suppressing the activity occludin expression and cleaving occludin through the metalloproteinase matrix. The dual-functionalized MWCNT improves oxaliplatin cytotoxicity for glioma cells considerably. Furthermore, the TBCNT@OXA nanosystem had an improved penetration ability relative to the blood–brain barrier and had strong anti-tumor activity on orthotopic glioma [92]. In vivo and in vitro results demonstrated that single-walled carbon nanotubes can be used as safe and excellent drug carriers in the treatment of Alzheimer disease or any diseases of the CNS [93]. The dose of the drug can be controlled to ensure lysosomal but not mitochondrial targeting. Doxorubicin (DOX) was targeted with PEGylated oxidized MWCNTs modified with angiopep-2 for brain glioma. Compared with doxorubicin, the anti-glioma effect of C6 cytotoxicity and the median survival period of glioma carrying mice showed a stronger anti-glioma effect of DOX-OMWNTs-PEG-ANG.

The biological safety of O-MWNTs-PEG-ANG assessed by using C6 cytotoxicity and BCEC, hematology analysis and CD68 immuno-histochemical study showed that O-MWNTs-PEG-ANG had low toxicity and good biocompatibility. The biological safety assessment by histopathological analysis showed lower cardiac toxicity than DOX. Based on this, it was concluded that O-MWNTs-PEG-ANG would be a promising dual-targeting carrier for delivering DOX for brain tumor treatment [94]. Similar investigations on functionalized SWCNTs conjugated with CpG (f-SWCNTs-CpG) were tested in mice bearing intracranial GL261 gliomas. Flow cytometry was used to test CNT-CpG absorption and antiglioma immune response. In both in vitro and intracranial gliomas, CNT-CpG showed increased CpG uptake. The production of proinflammatory cytokine potentiated by primary monocytes was observed. Significant eradication of intracranial GL261 gliomas was observed in half of the tumor-bearing mice after low-dose CNT-CpG single intracranial injection. Enhanced delivery CNTs into tumor-associated inflammatory cells can potentiate CpG immunopotency activity [95].

Amino f-CNT significantly improves aqueous dispersibility in 5% dextrose than compared to pristine CNTs. Silencing caspase-3 with in vivo RNA interference (RNAi) mediated by carbon nanotube could provide therapeutic opportunities against strokes [96]. The conjugation of two PiB derivative Gd^3^+ complexes, Gd(L2) and Gd(L3), to functionalized MWCNTs has shown significant enhancement in brain accumulation of the conjugates when compared to free metal complexes, and f-MWCNTs also acted as a potential carrier in theranostic applications of impermeable compounds for brain delivery with respect to crossing BBB [97].

By using the functionalized SWCNTs, “CAR” (SWCNT-PEGs-Lf) targeted dopamine (DA) delivery to the brain of mice with Parkinson’s disease (PD) was achieved for the treatment of Parkinsonism. Circulation time could be increased by PEG-coated SWCNTs, thus extending the concentration gradient of SWCNTs to the brain. The accumulation of lactoferrin-nanoparticles in the striatum was also shown. Thus, a dual modification of PEG and lactoferrin was applied to carry the drug to a specific site [98]. CNTs have the intrinsic ability to cross BBB in vitro and in vivo. In a study, researchers have developed an angiopep-2 (ANG) conjugated functionalized CNT for effective brain targeting. The inherent brain targeting of CNTs combined with ANG targeting favors future applications of glioma therapy [99].

### 8.3. CNTs in Gene Therapy

In a study related to gene therapy, the iC9 gene was used to induce apoptosis in human breast cancer cell line MCF-7. The iC9 gene was transferred through pyridine functionalized MWCNTs as an efficient delivery system. A combination of the introduced pf-MWCNT-delivered iC9 suicide gene therapy and chemotherapy was also performed. Reports showed that pyridine-functionalized multi-walled carbon nanotubes (pf-MWCNT)-delivered iC9 suicide gene therapy and is an effective strategy for killing cancer cells; combination therapy works even better than monotherapy due to overcoming cell cycle arrest resulting from chemical drugs [100]. A novel photoactivatable RNAi system was designed for targeted gene expression in tumor cells. It consists of stimulus-responsive nanocarrier polyetherimide-modified SWCNTs (PEI-SWCNTs) and Hsp 70B’-promoter-driven RNAi vector (pHSP-shT). Heating PEI-SWCNTs in MCF-7 cells triggered gene knockdown that targeted human telomerase reverse transcriptase via RNAi in the near infrared field (NIR). The study showed that the combined effect of gene therapy and photothermal therapy of the photoactivatable RNAi system showed significant antitumor activity [101]. Due to the targeted delivery of Bcl-xL-specific shRNA and doxorubicinin, synergistic tumor cell death was achieved. For the delivery of shRNA, SWCNT was linked to polyethyleneimine through a PEG linker. It was then conjugated to AS1411 aptamer as the nucleolin ligand in order to target the system relative to cancer cells. Together with a small quantity of doxorubicin, the system exhibited high tumoricidal activity [102]. A chitosan complexed SWCNT was developed for chloroplast transformation in some plants. CS-SWCNT utilizes the lipid exchange envelope penetration mechanism. CS-SWCNTs can effectively deliver plasmid DNA to the chloroplast of different plants. This nanoparticle-based transgene delivery offers practical advantages over conventional genetic engineering techniques [103]. Functionalized CNTs are better candidates for delivering plasmid DNA into non-model plant species, and these have resulted in high levels of protein expression without transgene integration. PEI modified SWCNTs can be used for effective plasmid DNA loading and delivery [104].

### 8.4. CNTs in Phototherapy

SWCNT was modified with hyaluronic acid for obtaining biocompatible water-soluble carriers for the targeted therapy of tumors. In laser irradiation fields, hyaluronic acid-modified SWCNTs generated heat due to photothermal sensitivity; hence, the system can be used for combined drug therapy and photothermal therapy. The system can also be used for the delivery of photothermal therapeutic agents [105]. In order to increase the anti-cancer checkpoint inhibitor’s efficacy, such as CTLA-4 antibodies, glycosylated chitosan (GC) modified SWCNTs were used in metastatic mammary tumors. Irradiation of laser after administration of GC-SWCNTs achieved local ablation via photothermal therapy (PTT). The combined effect of PTT, GC-SWCNT and CTLA-4 antibodies effectively inhibited tumors and metastasis [106].

Another study evaluated phototherapy and antibacterial effects of polypyrrole modified CNTs on *Pseudomonas aeruginosa*. At the NIR field, polypyrrole modified CNTs increased the temperature and induced photokilling of *P. aeruginosa* compared to the dark field; the NIR field induces a 70% killing rate, ROS generation, protein and nucleic acid leakage from *P. aeruginosa* [107]. A long circulating SWCNT complex was fabricated by using a new SWCNT dispersion agent, i.e., Evans blue. The complex was coupled with a fluorescent photosensitive albumin, chlorine e6 (Ce6). Synergistic Photodynamic therapy (PDT) and photothermal therapy (PTT) were obtained with an albumin/Ce6 loaded EB/SWCNT-based delivery system. Compared to PDT or PIT alone, the combined effect can prevent the recurrence of tumor cells [108]. CNTs have optical absorbance in the visible and NIR regions. Hence, it is a good candidate for photothermal therapy. Oxidized MWCNTs modified PEG with laser irradiation and showed a remarkable reduction in melanoma tumor size compared to laser therapy alone [109].

### 8.5. CNTs in Antiviral Therapy

The antiviral activities of highly hydrophilic and dispersible carboxylated MWCNTs and MWCNTs with two antiviral drugs newly synthesized have been studied. The newly synthesized drugs (CHI415 and CHI360) belong to non-nucleoside reverse transcriptase and lamivudine used for HIV-treatment. Study reports suggest that the two physical properties, hydrophilicity and dispersibility, are the key factors that control antiviral activity. The oxidized MWCNTs and MWCNTs with CHI360 showed more antiviral activity due to their high hydrophilicity and dispersibility [110]. A microfluidic platform was developed by Yeh et al. consisting of CNT arrays with differential filtration abilities for the rapid enrichment and detection of viruses. After virus capture and detection, the viruses remain viable and becomes purified in a device that allows further characterizations. With 70% enrichment enhancement, virus capture took only a few minutes [111].

### 8.6. CNTs in Antibacterial Therapy

Amine functionalized porphyrin conjugated SWCNTs were used in novel antimicrobial therapy by using visible light. This antimicrobial photodynamic therapy is a method devoid of any antibiotics. In the presence of visible light, the porphyrin conjugated CNTs result in cell membrane damage [112]. The antimicrobial activity of a different type of CNT (single-walled, double-walled and multi-walled) was studied on Gram-negative *Pseudomonas aeruginosa* and Gram-positive *Staphylococcus aures* and *Candida albicans*. The CNTs network attracted the pathogen through van der Waals forces. The killing effect is mainly due to the wrapping of CNTs over the pathogens rather than the piercing effect [113]. Silver coated SWCNTs (Ag-SWCNTs) possess a broad spectrum of antimicrobial activity. In order to decrease the toxicity of bare Ag-MWCNTs to human cells, PEGylated systems were developed and used to evaluate antibacterial activity. PEGylation renders Ag-SWCNTs non-toxic to eukaryotic cells. Studies showed that both nanocomposites damaged the cell membrane, followed by cell lysis [114]. In a study, the antibacterial activity of ionic and non-ionic surfactant conjugated MWCNTs against *E. coli* growth was studied. The study reported that cationic surfactant coated MWCNTs were more toxic to Gram-negative *E. coli*. Anionic surfactant coated MWCNTs are more toxic than non-ionic coated MWCNTs due to the cooperated toxicity of the surfactant [115]. Moreover, researchers created a bifunctional (both catalytic and antibacterial) novel nanocomposite with SWCNTs, Ag and Fe3O4 for water purification. The nanocomposite showed high antibacterial activity against Gram-negative and Gram-positive organisms, even in the presence of dissolved organic matters. However, the study reported that an increase in dissolved organic matters decreased the antibacterial activity of the nanocomposite [116].

### 8.7. CNTs in Antifungal Therapy

The antifungal activity of functional group attached MWCNTs was studied in plant pathogenic fungi *Fusarium graminearum*. MWCNT with –OH, –NH_2_ and –COOH fictionalization showed an inhibitory effect on spore elongation and germination of fungi. Functionalized MWCNTs reduced germination rate to 18.2%, which was three times lower than non-functionalized MWCNTs [117]. Moreover, the antifungal activity of various nanoparticles on a notorious fungal pathogen, *Botrytis cinerea*, on rose petals was studied. MWCNTs, fullerene and reduced graphene oxide were separately added to water agar plates at different concentrations (50 and 200 mg/L). The study reported that at 200 mg/L concentration, MWCNTs showed inhibitory on fungi on rose petals [118]. Another study has evaluated the amino acid fictionalization of MWCNTs for antifungal activity. The study results from 10 fungal species showed that arginine functionalized and lysine functionalized MWCNTs exhibited higher antifungal activities than pristine MWCNTs. The higher antifungal activity is due to the enhanced positive charge after fictionalization. Arginine functionalized MWCNTs showed slightly higher activity due to its more positive charge than compared to lysine [119].

### 8.8. Other Biomedical Applications

CNTs based devices are utilized in tissue engineering and also in the treatment of stem cell-based therapeutics, including neuronal regeneration, muscle, myocardial therapy and bone formation [120]. CNTs have specific optical properties such as high absorption rate, photoluminescence and strong Raman shift, thereby demonstrating that CNTs are excellent agents for bioimaging and detection [121]. Generally, the immune system rejects foreign substances and helps in eliminating these substances from the body. Similarly, implants also become rejected by the body and causes post administration pain as the body treats that implant as a foreign body. However, it can be minimized by using CNTs as CNTs will attach to other proteins and amino acids in order to avoid any rejection by the body. Due to their antioxidant properties, CNTs can be used as a preservative in different formulations prone to oxidation, such as anti-aging in cosmetics. CNTs loaded with zinc oxide are used as an emollient dermatologic to stop the oxidization of necessary skin elements [122]. CNTs that are encapsulated within the macromolecule or protein will have fluorescence abilities within the presence of specific biomolecules, and that might be tested as an implantable biosensor. Nanocapsules loaded with radioisotope enzymes and magnetic materials are used as biosensors [123].

**Figure 8:**
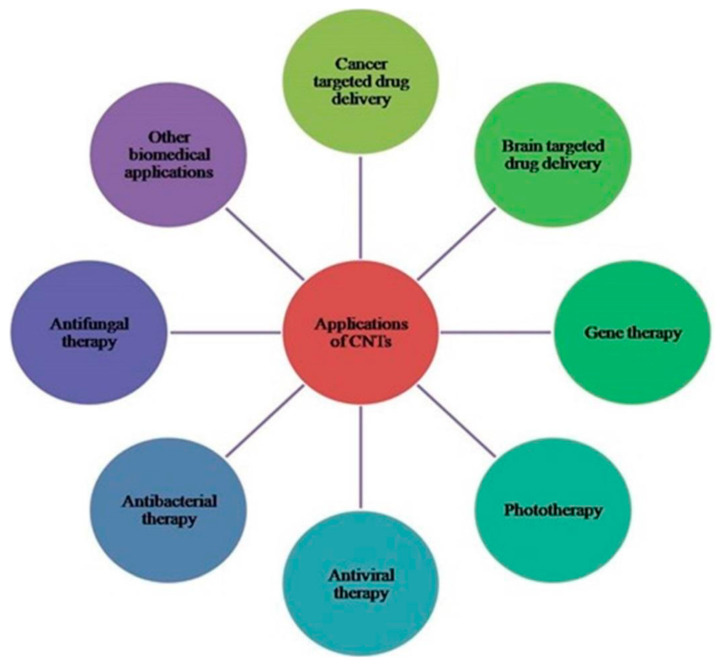
Diverse applications of CNTs.

Motors and nano sized robots with CNTs are employed in studying cells and biological systems [124]. Single stranded deoxyribonucleic acid that is unwound winds around SWCNTs by connecting its unique nucleotides, causing changes in its electrostatic property and thereby creating its potential use in therapeutics (polymerase chain reaction) and in diagnostics. Wrappings of CNTs by single stranded deoxyribonucleic acid that may be sequence-dependent may be utilized in deoxyribonucleic acid analysis. CNTs are utilized in biotechnology to manipulate different genes and atoms within the development of bioimaging genomes, tissue engineering and proteomics [125]. CNTs have unique cylindrical or hollow structures, and this property can be applied for the treatment of genetic disorder by choosing it as a perfect carrier. Tubular or hollow natured CNTs have also proven themselves as a vector in gene therapy. CNTs complexed with DNA release DNA before it is destroyed by the defense system of cells, which results in boosting transfection significantly. CNTs are also used in the treatment of respiratory diseases such as asthma and bronchitis caused by respiratory syncytial virus (RSV), and therapy is performed by combining CNTs along with gene sequence slicing technologies. RNA fragments are able to inhibit proteins; thus, they can be encapsulated in CNTs and can be administered in the form of nasal sprays or drops that may inhibit further growth of viruses [126]. By immobilization of streptavidin macromolecule on CNTs with 1-pyrene saturated fatty acid and succinimidyl ester, various antigens can be attached to the surface of CNTs; thus, they can act as a source of antigen in vaccines [127]. Consequently, the traditional method of utilizing dead microorganisms as an antigen source can be avoided by using CNTs. Figure 8 represents the diverse applications of CNTs.

## 9. Patents on CNTs

Many researchers throughout the world have published patents on CNTs. In one of the inventions, CNTs are dissolved in organic solutions in order to render carbon nanotubes soluble, and it is carried out by attaching an aliphatic carbon chain containing aromatic residues [128]. In other inventions, protrusions are formed on a substrate such as silicon and quartz glass (where the substrate is heated), followed by coating catalytic metals of transition elements such as nickel, iron and cobalt on the substrate. CNTs are grown by microwave-plasma enhanced chemical vapor deposition or hot-filament chemical vapor deposition under applications of negative voltage to the substrate. In these methods, CNTs can be selectively grown from the apex of protrusions. As a substrate, a silicon probe for scanning probe microscopy (SPM) can be used. The carbon nanotube probe can be applied to high resolution SPM probe for imaging a precise topographic image [129]. In one of the inventions, a CNT product has been formed from a metallic catalytic particle, and SWCNTs were deposited, ideally containing at least one metal (Ni, Ru, Co, Ir, Pd, Rh and Pt) from Group VIII and at least one metal from Group VIb (including Cr, Mo and W). A CNT product is formed preferably at sufficient temperatures by exposing the metallic catalytic particle to carbon-containing gas in order to form single-walled nanotubes [130]. Moreover, the manufacturing method for forming open-ended CNTs is described. Different steps involved in manufacturing are mentioned. The steps begin with the first step of “providing a substrate having a catalyst layer formed thereon, followed by placing the substrate in a reaction chamber. It is then followed by introducing a carbon source gas containing carbon element into reaction chamber CNTs growth. Finally, it is then followed by promptly reducing a concentration of carbon source gas when the growth of CNTs in process thereby ceasing the growth of the CNTs instantly and separating the CNTs from catalyst layer” [131]. In a different study, the method for making graphene nanoribbons (GNRs) was mentioned. It was achieved by controlled unzipping of structures such as CNTs by etching nanotubes (argon plasma etching) partly embedded in a polymer film. GNRs were found to have smooth edges with narrow width (2–20 nm) distributions, and high quality GNRs were revealed by electrical transport measurements and Raman spectroscopy [132]. Another innovation was related to fullerene CNTs with a cylindrical wall that comprise a double layer of carbon atoms. Production methods and the applications of DWCNTs were also mentioned [133]. Moreover, one of the studies was related to producing CNTs by providing finely divided substrate particles that have substantially smooth faces and wherein the surface is smooth over an order of size of catalyst material clusters, i.e., from 0.5 to 100 nm in dimension [134]. In an attempt, the composition was produced and consisted of a mixture of CNTs possessing bi-modal size distribution. It was produced in the presence of a catalyst by reducing carbon oxides with a reducing agent, and the resulting mixture included a primary population of MWCNTs with diameters more than 40 nm and a secondary population of SWCNTs with diameters less than 30 nm [135]. In addition, there is a report about producing SWCNTs with different steps, beginning with the first step of continuously providing substrate particles. This step is then followed by providing a transition decomposable metal oxalate or formate on substrate particles in order to provide a the transition metal under a non-reducing atmosphere, which allows CNT formation. It is then followed by fluidizing the substrate particles with a flow of gaseous carbon source and then heating the transition metal oxalate or formate on substrate particles [119]. A method for CNT purification was also reported, which is a purification method that included providing carbon nanotubes, depositing a mask and/or selectively removing a portion of the mask and optionally including removing a subset of the CNTs and/or removing the remaining mask [136]. A list of recent patents is provided in Table 1.

## 10. Pharmacokinetics and Metabolism of CNTs

Several research studies have investigated the pharmacokinetics and metabolism of different types of CNTs, and some reviews have recently been published [76,137,138]. The pharmacokinetics and bio-distribution of nanoparticles are based on their physicochemical characteristics such as solubility, aggregation, shape, surface functionalization and chemical composition. The literature shows that two studies were performed in mice on the bio-distribution of water-soluble CNTs (SWCNT or/and MWCNTs) [137,138,139]. Neither of these studies reported toxic side effects or death. In both studies, ^111^Indium or ^125^Iodine have been used as radiotracers for monitoring its biodistribution in mice [139,140]. In the first study, ^125^I-hydroxylated-SWCNT (^125^I-SWCNT-OH) where investigated via different routes (oral, SC, IV or intraperitoneal). In this analysis, CNT bio-distribution was not influenced significantly by the route, and ^125^I-SWCNT-OH was quickly distributed all over the body with 6 percent of unchanged nanotubes excreted in feces and 94 percent in urine [137,139], respectively. Two types of ^111^Indium-functional SWCNT or MWCNT were only used for mice (IV route) for the second study. For both forms of functional CNT, which showed an affinity for skin, muscles, kidneys, blood and bone 30 min after administration, the biodistribution profiles that were obtained were very similar [139,140]. All the types of CNTs have been quickly removed from all tissues, and a maximum circulation of blood has been determined with a half-life of 3.5 h [139]. It has been observed that SWCNT and MWCNT are excreted through the renal pathway and found to be intact in excreted urine by TEM [138]. However, some researchers have recently shown that myeloperoxidase, an enzyme found in neutrophils of mice, can break down CNTs [141,142,143]. Their results are contrary to what was previously thought, which is that CNTs are not broken in the body. This behavior of how myeloperoxidase transforms CNTs into the water and carbon dioxide can be a big advantage for medicine and is, therefore, a breakthrough in nanotoxicology and nanotechnology, although this shows clearly that endogenous myeloperoxidase can break down CNT [142,143,144,145].

## 11. Conclusions

Nanotechnology is considered as one of the prime innovating branches of the science of the present century and is the most significant trend in research. It is creating remarkable impressions in different streams of research such as analytical tools, pharmaceutical sciences, medicine, electronics, industrial manufacturing processes, mechanical industry, etc., to list a few. Nanotechnology represents the production of novel materials of a size of 100 nm or even smaller. One such novel material or carrier in the field of nanotechnology is CNTs, which have attracted more attention in recent times. Their encouraging features that render them the nanocarriers of prime choice are that CNTs are very stable due to their unique and promising chemical, physical and mechanical properties. CNTs have also been greatly implied for site-specific drug delivery and drug release in a controlled manner. CNTs can also be loaded with a high amount of drugs due to their hollow tubular structure, and it can, in turn, result in increased efficiency of therapeutic molecules. It has been also well-established that CNTs can deliver therapeutic drugs and moieties into cancerous cells through endocytosis or penetration into the cell without causing apparent cell damage. Thus, they can also be used for augmented cancer therapy as a drug delivery agent or carrier for targeted drug delivery to cancers as well as in various allied cancer therapies.

Owing to their unique mechanical, electrical, chemical and physical characteristics, CNTs have gained a great deal of attention from researchers across all disciplines. Considering their high demand and applicability, CNTs are usually produced at a large-scale that results in high exposure to the general population either by indirect or direct modes. In recent times, this has prompted safety concerns on the effects of CNTs on human health and issues related to their toxicities. Although various rigorous research has been conducted in this direction and plenty of substantial experimental reports and data allied to CNTs toxicity at the cellular, molecular and animal levels are available, the derived conclusions do not align with each other, and several conflicts exist.

Although no one can deny the promising features and wide range of applications offered by CNTs, on the other hand, failure in reaching a consensus in CNTs toxicity limits easy and rapid industrial transition and clinical translation. Owing to critical limitations such as a high degree of toxicity, drug loading inadequacies, dose dumping risks, non-assurance of biodegradability and complete elimination, etc., undertaking clinical trials is a big conquest in and of itself. Furthermore, variability in loading capacities renders dose optimization much complicated. Hence, CNT-based delivery systems and therapeutics are still in the pipeline for FDA and other regulatory approvals. However, CNT functionalization offers a method of improving solubility and biocompatibility and, thus, leads towards newer avenues utilizing CNTs in the era of nanomedicine.

The methods applied to manipulate the surfaces of CNTs influence toxicity greatly. Hence, detailed material and structural characterization, mode and extent of uptake by the exposed cells and CNT eliminations from the administered body compartment along with the elimination mechanisms need to be more deeply explored. Ameliorating CNTs by functionalizing them before their biomedical application should be a recommended practice. With increasing demand and the large-scale production, there should be safety regulations and strategies undertaken in order to assess the allied risks and effects on employees who are exposed to CNTs. Setting in place standardized protocols for evaluating CNTs toxicity and categorizing their associated risks would be of great significance in accelerating their applications. Moreover, the hazards after CNT treatments should be assessed with conventional treatment modalities for finalizing safe doses and for comparing the relative benefits of available conventional regimens with CNT-based therapy. Continued focused efforts by scientists and researchers on CNTs should be encouraged, which would surely produce safer products that will enhance the existence and quality of the environment as well as human health. In a nutshell, CNTs and CNT-based drug delivery systems hold enormous potential and applicability in medical, biomedical, drug delivery and therapeutics and would be a centerpiece of research in the near future.

## Figures and Tables

**Table 1:** List of patents on CNTs.

Title of the Patent	Name of the Author(s)	Patent Number	Date of Publication
Method of solubilizing carbon nanotubes in organic solutions	Robert C. Haddon and Mark A. Hamon	US6531513B2	11 March 2003
Fabrication method of carbon nanotubes	Takahito Ono and Esashi Masayoshi	US20040036403A1	26 February 2004
CNTs product comprising single-walled carbon nanotubes	DE. Resasco, B Kitiyanan, JH. Harwell and W Alvarez	US6994907B2	7 February 2006
Method for manufacturing carbon nanotubes	Kai Liu, Kai-Li Jiang and Shou-Shan Fan	US7625544B2	1 December 2009
Narrow graphene nanoribbons from carbon nanotubes	Hongie Dai and Liying Jiao	US8236626B2	7 August 2012
Double-walled carbon nanotubes and methods for production and application	Alexander P. Moravsky and Raouf O. Loutfy	US8404209B2	26 March 2013
Method for producing carbon nanotubes and/or nanofibres	I Kinloch, C Singh and M Sebastian Peter Shaffer, Krzysztof K. K. Koziol and AH. Windle	EP1560790B1	22 February 2017
Methods of forming carbon nanotubes having a bimodal size distribution	Dallas B. Noyes	US9896341B2	20 February 2018
Cvd synthesis of carbon nanotubes	Milo Sebastian Peter Shaffer, Alan H. Windle, Brian F. G. The Masters Lodge Johnson, Junfeng Geng, Douglas Shephard and Charanjeet Singh	EP1558524B1	16 January 2019
Method for carbon nanotube purification	John Provine, Cara Beasley and Gregory Pitner	US20200216320A1	9 July 2020

## Data Availability

This study did not report any data.

## References

[1] Blay J.-Y., Cesne A.L., Alberti L., Ray-Coquart I. (2005). Targeted cancer therapies. Bull. Cancer.

[2] Bianco A., Kostarelos K., Prato M. (2005). Applications of carbon nanotubes in drug delivery. Curr. Opin. Chem. Biol..

[3] Wang X., Li Q., Xie J., Jin Z., Wang J., Li Y., Jiang K., Fan S. (2009). Fabrication of ultralong and electrically uniform single-walled carbon nanotubes on clean substrates. Nano Lett..

[4] Wang X., Yang L., Chen Z., Shin D.M. (2008). Application of nanotechnology in cancer therapy and imaging. CA Cancer J. Clin..

[5] Yang W., Thordarson P., Godding J.J., Ringer S.P., Braet F. (2007). Carbon nanotubes for biological and biomedical applications. Nanotechnology.

[6] Hilder T.A., Hill J.M. (2009). Modeling the loading and unloading of drugs into nanotubes. Small.

[7] Thordarson P., Droumaguet B.L., Velonia K. (2006). Well-defined protein–polymer conjugates—Synthesis and potential applications. Appl. Microbiol. Biotechnol..

[8] Chen X., Lee G.S., Zettl A., Bertozzi C.R. (2004). Biomimetic engineering of carbon nanotubes by using cell surface mucin mimics. Angew. Chem. Int. Ed. Engl..

[9] Pastorin G. (2009). Crucial functionalizations of carbon nanotubes for improved drug delivery: A valuable option?. Pharm. Res..

[10] Prato M., Kostarelos K., Bianco A. (2008). Functionalized carbon nanotubes in drug design and discovery. Acc. Chem. Res..

[11] Bahr J.L., Tour J.M. (2002). Covalent chemistry of single-wall carbon nanotubes. J. Mater. Chem..

[12] Zhu H.W., Xu C.L., Wu D.H., Wei B.Q., Vajtai R., Ajayan P.M. (2002). Direct synthesis of long single-walled carbon nanotube strands. Science.

[13] Kostarelos K., Lacerda L., Partidos C.D., Prato M., Bianco A. (2005). Carbon nanotube-mediated delivery of peptides and genes to cells: Translating nanobiotechnology to therapeutics. J. Drug Deliv. Sci. Technol..

[14] Tran P.A., Zhang L., Webster T.J. (2009). Carbon nanofibers and carbon nanotubes in regenerative medicine. Adv. Drug Deliv. Rev..

[15] Yang S.-T., Wang X., Jia G., Gu Y., Wang T., Nie H., Ge C., Wang H., Liu Y. (2008). Long-term accumulation and low toxicity of single-walled carbon nanotubes in intravenously exposed mice. Toxicol. Lett..

[16] Neves L.F., Krais J.J., Van Rite B.D., Ramesh J., Resasco D.E., Harrison R.G. (2013). Targeting single-walled carbon nanotubes for the treatment of breast cancer using photothermal therapy. Nanotechnology.

[17] Lacerda L., Bianco A., Prato M., Kostarelos K. (2006). Carbon nanotubes as nanomedicines: From toxicology to pharmacology. Adv. Drug Deliv. Rev..

[18] Saito N., Usui Y., Aoki K., Narita N., Shimizu M., Ogiwara N., Nakamura K., Ishigaki N., Kato H., Taruta S. (2008). Carbon nanotubes for biomaterials in contact with bone. Curr. Med. Chem..

[19] Beg S., Rizwan M., Sheikh A.M., Hasnain M.S., Anwer K., Kohli K. (2011). Advancement in carbon nanotubes: Basics, biomedical applications and toxicity. J. Pharm. Pharmacol..

[20] Ebbesen T., Ajayan P. (1992). Large-scale synthesis of carbon nanotubes. Nature.

[21] Rao C., Govindaraj A. (2003). Carbon nanotubes from organometallic precursors. Advances In Chemistry: A Selection of CNR Rao’s Publications (1994–2003).

[22] Murakami T., Fan J., Yudasaka M., Iijima S., Shiba K. (2006). Solubilization of single-wall carbon nanohorns using a PEG—Doxorubicin conjugate. Mol. Pharm..

[23] Journet C., Maser W.K., Bernier P., Loiseau A., de la Chapelle M.L., Lefrant S., Deniard P., Lee R., Fischer J.E. (1997). Large-scale production of single-walled carbon nanotubes by the electric-arc technique. Nature.

[24] Yamaguchi T., Bandow S., Iijima S. (2004). Synthesis of carbon nanohorn particles by simple pulsed arc discharge ignited between pre-heated carbon rods. Chem. Phys. Lett..

[25] Dai H., Rinzler A.G., Nikolaev P., Thess A., Colbert D.T., Smalley R.E. (1996). Single-wall nanotubes produced by metal-catalyzed disproportionation of carbon monoxide. Chem. Phys. Lett..

[26] Vander Wal R.L., Berger G.M., Hall L.J. (2002). Single-walled carbon nanotube synthesis via a multi-stage flame configuration. J. Phys. Chem. B.

[27] Shah K.A., Tali B.A. (2016). Synthesis of carbon nanotubes by catalytic chemical vapour deposition: A review on carbon sources, catalysts and substrates. Mater. Sci. Semicond. Proc..

[28] Nikolaev P., Bronikowski M.J., Bradley R.K., Rohmund F., Colbert D.T., Smith K.A., Smalley R.E. (1999). Gas-phase catalytic growth of single-walled carbon nanotubes from carbon monoxide. Chem. Phys. Lett..

[29] Resasco D.E., Alvarez W.E., Pompeo F., Balzano L., Herrera J.E., Kitiyanan B., Borgna A. (2002). A scalable process for production of single-walled carbon nanotubes (SWNTs) by catalytic disproportionation of CO on a solid catalyst. J. Nanopart. Res..

[30] Terrones M. (2003). Science and technology of the twenty-first century: Synthesis, properties, and applications of carbon nanotubes. Ann. Rev. Mater. Res..

[31] Mittal G., Dhand V., Rhee K.Y.I., Kim H.J., Jung D.H. (2015). Carbon nanotubes synthesis using diffusion and premixed flame methods: A review. Carbon Lett..

[32] Li H., Zhang N., Hao Y., Wang Y., Jia S., Zhang H., Zhang Y., Zhang Z. (2014). Formulation of curcumin delivery with functionalized single-walled carbon nanotubes: Characteristics and anticancer effects in vitro. Drug Deliv..

[33] Zhao Y., Yang L., Chen S., Wang X., Ma Y., Wu Q., Jiang Y., Qian W., Hu Z. (2013). Can boron and nitrogen co-doping improve oxygen reduction reaction activity of carbon nanotubes?. J. Am. Chem. Soc..

[34] Datsyuk V., Kalyva M., Papagelis K., Parthenios J., Tasis D., Siokou A., Kallitsis I., Galiotis C. (2008). Chemical oxidation of multiwalled carbon nanotubes. Carbon.

[35] Chin S.-F., Baughman R.H., Dalton A.B., Dieckmann G.R., Draper R.K., Mikoryak C., Musselman I.H., Poenitzsch V.Z., Xie H., Pantano P. (2007). Amphiphilic helical peptide enhances the uptake of single-walled carbon nanotubes by living cells. Exp. Biol. Med..

[36] Dang Z.-M., Wang L., Zhang L.-P. (2006). Surface functionalization of multiwalled carbon nanotube with trifluorophenyl. J. Nanomater..

[37] Pantarotto D., Briand J.P., Prato M., Bianco A. (2004). Translocation of bioactive peptides across cell membranes by carbon nanotubes. Chem. Comm..

[38] Raffa V., Ciofani G., Vittorio O., Riggio C., Cuschieri A. (2010). Physicochemical properties affecting cellular uptake of carbon nanotubes. Nanomedicine.

[39] Porter A.E., Gass M., Muller K., Skepper J.N., Midgley P.A., Welland M. (2007). Direct imaging of single-walled carbon nanotubes in cells. Nat. Nanotechnol..

[40] Cai D., Mataraja J.M., Qin J.H., Huang Z., Chiles T.C., Carnahan D., Kempa K., Ren Z. (2005). Highly efficient molecular delivery into mammalian cells using carbon nanotube spearing. Nat. Methods.

[41] Klumpp C., Kostarelos K., Prato M., Bianco A. (2006). Functionalized carbon nanotubes as emerging nanovectors for the delivery of therapeutics. Biochim. Biophys. Acta.

[42] Kam N.W.S., O’Connell M., Wisdom J.A., Dai H. (2005). Carbon nanotubes as multifunctional biological transporters and near-infrared agents for selective cancer cell destruction. Proc. Natl. Acad. Sci. USA.

[43] Kam N.W.S., Liu Z., Dai H. (2006). Carbon nanotubes as intracellular transporters for proteins and DNA: An investigation of the uptake mechanism and pathway. Angew. Chem. Int. Ed. Engl..

[44] Chen X., Kis A., Zettl A., Bertozzi C.R. (2007). A cell nanoinjector based on carbon nanotubes. Proc. Natl. Acad. Sci. USA.

[45] Dhar S., Liu Z., Thomale J., Dai H., Lippard J. (2008). Targgeted sungle-wall carbon nanotube-mediated Pt(IV) prodrug delivery using folate as a homing device. J. Am. Chem. Soc..

[46] Esfandiary E., Valiani A., Hashemibeni B., Moradi I., Narimani M. (2014). The evaluation of toxicity of carbon nanotubes on the human adipose-derived-stem cells in-vitro. Adv. Biomed. Res..

[47] Davoren M., Herzog E., Casey A., Cottineau B., Chambers D., Byrne H.J., Lyng F.M. (2007). In vitro toxicity evaluation of single walled carbon nanotubes on human A549 lung cells. Toxicol. Vitr..

[48] Belyanskaya L., Manser P., Spohn P., Bruinink A., Wick P. (2007). The reliability and limits of the MTT reduction assay for carbon nanotubes-cell interaction. Carbon.

[49] Cui D., Tian F., Ozkan C.S., Wang M., Gao H. (2005). Effect of single wall carbon nanotubes on human HEK293 cells. Toxicol. Lett..

[50] Shvedova A.A., Castranova V., Kisin E.R., Schwegler-Berry D., Murray A.R., Gandelsman V.Z., Maynard A., Baron P. (2003). Exposure to carbon nanotube material: Assessment of nanotube cytotoxicity using human keratinocyte cells. J. Toxicol. Environ. Health Part A.

[51] Kagan V.E., Tyurina Y.Y., Tyurin V.A., Konduru N.V., Potapovich A.I., Osipov A.N., Kisin E.R., Schwegler-Berry D., Mercer R., Castranova V. (2006). Direct and indirect effects of single walled carbon nanotubes on RAW 264.7 macrophages: Role of iron. Toxicol. Lett..

[52] Magrez A., Kasas S., Salicio V., Pasquier N., Seo J.W., Celio M., Catsicas S., Schwaller B., Forro L. (2006). Cellular toxicity of carbon-based nanomaterials. Nano Lett..

[53] Bottini M., Bruckner S., Nika K., Bottini N., Bellucci S., Margini A., Bergamaschi A., Mustelin T. (2006). Multi-walled carbon nanotubes induce T lymphocyte apoptosis. Toxicol. Lett..

[54] Donaldson K., Stine V., Tran C.L., Kreyling W., Borm P.J.A. (2004). Nanotoxicology. Occup. Environ. Med..

[55] Cheng C., Muller K.H., Koziol K.K.K., Skepper J.N., Midgley P.A., Welland M.E., Porter A.E. (2009). Toxicity and imaging of multi-walled carbon nanotubes in human macrophage cells. Biomaterials.

[56] Sayes C.M., Liang F., Hudson J.L., Mendez J., Guo W., Beach J.M., Moore V.C., Doyle C.D., West J.L., Billups W.E. (2006). Functionalization density dependence of single-walled carbon nanotubes cytotoxicity in vitro. Toxicol. Lett..

[57] Thurnherr T., Brandenberger D., Fischer K., Diener L., Manser P., Maeder-Althaus X., Kaiser J.P., Krug H.F., Rothen-Rutishauser B., Wick P. (2011). A comparison of acute and long-term effects of industrial multiwalled carbon nanotubes on human lung and immune cells in vitro. Toxicol. Lett..

[58] Schipper M.L., Nakayama-Ratchford N., Davis C.R., Kam N.W.S., Chu P., Liu Z., Sun X., Dai H., Gambhir S.S. (2008). A pilot toxicology study of single-walled carbon nanotubes in a small sample of mice. Nat. Nanotechnol..

[59] Liu Z., Davis C., Cai W., He L., Chen X., Dai H. (2008). Circulation and long-term fate of functionalized, biocompatible single-walled carbon nanotubes in mice probed by Raman spectroscopy. Proc. Natl. Acad. Sci. USA.

[60] Ema M., Takehara H., Naya M., Kataura H., Fujita K., Honda K. (2017). Length effects of single-walled carbon nanotubes on pulmonary toxicity after intratracheal instillation in rats. J. Toxicol. Sci..

[61] Fujita K., Fukuda M., Endoh S., Maru J., Kato H., Nakamura A., Shinohara N., Uchino K., Honda K. (2015). Size effects of single-walled carbon nanotubes on in vivo and in vitro pulmonary toxicity. Inhal. Toxicol..

[62] Pacurari M., Yin X.J., Zhao J., Ding M., Leonard S.S., Schwegler-Berry D., Ducatman B., Sbarra D., Hoover M.D., Castranova V. (2008). Raw single-wall carbon nanotubes induce oxidative stress and activate MAPKs, AP-1, NF-kappaB, and Akt in normal and malignant human mesothelial cells. Environ. Health Perspect..

[63] Gatoo M.A., Naseem S., Arfat M.Y., Dar A.M., Qasim K., Zubair S. (2014). Physicochemical properties of nanomaterials: Implication in associated toxic manifestations. BioMed Res. Int..

[64] Kostal J., Voutchkova-Kostal A., Anastas P.T., Zimmerman J.B. (2015). Identifying and designing chemicals with minimal acute aquatic toxicity. Proc. Natl. Acad. Sci. USA.

[65] Lee K.J., Browning L.M., Nallathamby P.D., Xu X.H. (2013). Study of charge-dependent transport and toxicity of peptide-functionalized silver nanoparticles using zebrafish embryos and single nanoparticle plasmonic spectroscopy. Chem. Res. Toxicol..

[66] Bozich J.S., Lohse S.E., Torelli M.D., Murphy C.J., Hamers R.J., Klaper R.D. (2014). Surface chemistry, charge and ligand type impact the toxicity of gold nanoparticles to Daphnia magna. Environ. Sci. Nano.

[67] Gilbertson L.M., Melnikov F., Wehmas L.C., Anastas P.T., Tanguay R.L., Zimmerman J.B. (2016). Toward safer multi-walled carbon nanotube design: Establishing a statistical model that relates surface charge and embryonic zebrafish mortality. Nanotoxicology.

[68] Rizzo L.Y., Golombek S.K., Mertens M.E., Pan Y., Laaf D., Broda J., Jayapaul J., Mockel D., Subr V., Kiessling F. (2013). In vivo nanotoxicity testing using the zebrafish embryo assay. J. Mater. Chem. B.

[69] Luo J., Solimini N.L., Elledge S.J. (2009). Principles of cancer therapy: Oncogene and non-oncogene addiction. Cell.

[70] Sinha R., Kim G.J., Nie S., Shin D.M. (2006). Nanotechnology in cancer therapeutics: Bioconjugated nanoparticles for drug delivery. Mol. Cancer Ther..

[71] Prakash S., Malhotra M., Shao W., Tomaro-Duchesneau C., Abbasi S. (2011). Polymeric nanohybrids and functionalized carbon nanotubes as drug delivery carriers for cancer therapy. Adv. Drug Deliv. Rev..

[72] Carmeliet P., Jain R.K. (2000). Angiogenesis in cancer and other diseases. Nature.

[73] Maeda H. (2001). SMANCS and polymer-conjugated macromolecular drugs: Advantages in cancer chemotherapy. Adv. Drug Deliv. Rev..

[74] Matsumura Y., Maeda H. (1986). A new concept for macromolecular therapeutics in cancer chemotherapy: Mechanism of tumoritropic accumulation of proteins and the antitumor agent smancs. Cancer Res..

[75] Hillebrenner H., Buyukserin F., Kang M., Mota M.O., Stewart J.D., Martin C.R. (2006). Corking nano test tubes by chemical self-assembly. J. Am. Chem. Soc..

[76] Zhang W., Zhang Z., Zhang Y. (2011). The application of carbon nanotubes in target drug delivery systems for cancer therapies. Nanoscale Res. Lett..

[77] Madani S.Y., Naderi N., Dissanayake O., Tan A., Seifalian A.M. (2011). A new era of cancer treatment: Carbon nanotubes as drug delivery tools. Int. J. Nanomed..

[78] Garse H., Vij M., Yamgar M., Kadam V., Hirlekar R. (2010). Formulation and evaluation of a gastroretentive dosage form of labetalol hydrochloride. Arch. Pharm. Res..

[79] Yan Y., Wang R., Hu Y., Sun R., Song T., Shi X., Yin S. (2018). Stacking of doxorubicin on folic acid-targeted multiwalled carbon nanotubes for in vivo chemotherapy of tumors. Drug Deliv..

[80] Jawahar N., De A., Jubee S., Reddy E.S. (2020). Folic acid-conjugated raloxifene hydrochloride carbon nanotube for targeting breast cancer cells. Drug Dev. Res..

[81] Wang D., Ren Y., Shao Y., Yu D., Meng L. (2017). Facile preparation of doxorubicin-loaded and folic acid-conjugated carbon nanotubes@ poly (N-vinyl pyrrole) for targeted synergistic chemo–Photothermal cancer treatment. Bioconj. Chem..

[82] Kam N.W., Dai H. (2005). Carbon nanotubes as intracellular protein transporters: Generality and biological functionality. J. Am. Chem. Soc..

[83] Kostarelos K., Larerda L., Pastorin G., Wu W., Wieckowski S., Luangsivilay J., Godefroy S., Pantarotto D., Braind J.-P., Muller S. (2007). Cellular uptake of functionalized carbon nanotubes is independent of functional group and cell type. Nat. Nanotechnol..

[84] Brenner B.M., Hostetter T.H., Humes H.D. (1978). Glomerular permselectivity: Barrier function based on discrimination of molecular size and charge. Am. J. Physiol..

[85] Yang F., Fu D.L., Long J., Ni Q.X. (2008). Magnetic lymphatic targeting drug delivery system using carbon nanotubes. Med. Hypothes.

[86] Zhang L., Xia J., Zhao Q., Liu L., Zhang Z. (2010). Functional graphene oxide as a nanocarrier for controlled loading and targeted delivery of mixed anticancer drugs. Small.

[87] Wu W., Weickowski S., Pastorin G., Benincasa M., Klumpp C., Braind J.P., Gennaro R., Prato M., Bianco A. (2005). Targeted delivery of amphotericin B to cells by using functionalized carbon nanotubes. Angew. Chem. Int. Ed. Engl..

[88] Balakumar K., Rajkumar M., Raghavan C.V. (2012). Carbon nanotubes: A versatile technique for drug delivery. Int. J. Nanomater. Biostruc..

[89] Wong B.S., Yoong S.L., Jagusiak A., Panczyk T., Ho H.K., Ang W.H., Pastorin G. (2013). Carbon nanotubes for delivery of small molecule drugs. Adv. Drug Deliv. Rev..

[90] Costa P.M., Bourgognon M., Wang J.T.W., Al-Jamal K.T. (2016). Functionalised carbon nanotubes: From intracellular uptake and cell-related toxicity to systemic brain delivery. J. Control. Release.

[91] Tan J.M., Foo J.B., Fakurazi S., Hussein M.Z. (2015). Release behaviour and toxicity evaluation of levodopa from carboxylated single-walled carbon nanotubes. Beilstein J. Nanotechnol..

[92] You Y., Wang N., He L., Shi C., Zhang D., Liu Y., Luo L., Chen T. (2019). Designing dual-functionalized carbon nanotubes with high blood–brain-barrier permeability for precise orthotopic glioma therapy. Dalton Trans..

[93] Yang Z., Zhang Y., Yang Y., Sun L., Han W., Li H., Wang C. (2010). Pharmacological and toxicological target organelles and safe use of single-walled carbon nanotubes as drug carriers in treating Alzheimer disease. Nanomedicine.

[94] Ren J., Shen S., Wang D., Xi Z., Guo L., Pang Z., Qian Y., Sun X., Jiang X. (2012). The targeted delivery of anticancer drugs to brain glioma by PEGylated oxidized multi-walled carbon nanotubes modified with angiopep-2. Biomaterials.

[95] Zhao D., Alizadeh D., Zhang L., Liu W., Farrukh O., Manuel E., Diamond D.J., Badie B. (2011). Carbon nanotubes enhance CpG uptake and potentiate antiglioma immunity. Clin. Cancer Res..

[96] Al-Jamal K.T., Gherardini L., Bardi G., Nunes A., Guo C., Bussy C., Herrero M.A., Bianco A., Prato M., Kostarelos K. (2011). Functional motor recovery from brain ischemic insult by carbon nanotube-mediated siRNA silencing. Proc. Natl. Acad. Sci. USA.

[97] Costa P.M., Wang J.T.W., Morfin J.F., Khanum T., To W., Sosabowski J., Toth E., Al-Jamal K.T. (2018). Functionalised carbon nanotubes enhance brain delivery of amyloid-targeting Pittsburgh Compound B (PiB)-derived ligands. Nanotheranostics.

[98] Guo Q., You H., Yang X., Lin B., Zhu Z., Lu Z., Li X., Zhao Y., Mao L., Shen S. (2017). Functional single-walled carbon nanotubes ‘CAR’for targeting dopamine delivery into the brain of parkinsonian mice. Nanoscale.

[99] Kafa H., Wang J.T.W., Rubio N., Klippstein R., Costa P.M., Hassan H.A.F.M., Sosabowski J.K., Bansal S.S., Preston J.E., Abbott N.J. (2016). Translocation of LRP1 targeted carbon nanotubes of different diameters across the blood–brain barrier in vitro and in vivo. J. Control. Release.

[100] Mohseni-Dargah M., Akbari-Birgani S., Madadi Z., Saghatchi F., Kaboudin B. (2019). Carbon nanotube-delivered iC9 suicide gene therapy for killing breast cancer cells in vitro. Nanomedicine.

[101] Ren X., Lin J., Wang X., Liu X., Meng E., Zhang R., Sang Y., Zhang Z. (2017). Photoactivatable RNAi for cancer gene therapy triggered by near-infrared-irradiated single-walled carbon nanotubes. Int. J. Nanomed..

[102] Taghavi S., Nia A.H., Abnous K., Ramezani M. (2017). Polyethylenimine-functionalized carbon nanotubes tagged with AS1411 aptamer for combination gene and drug delivery into human gastric cancer cells. Int. J. Pharm..

[103] Kwak S.-Y., Lew T.T.S., Sweeney C.J., Koman V.B., Wong M.H., Bohmert-Tatarev K., Snell K.D., Seo J.S., Chua N.H., Strano M.S. (2019). Chloroplast-selective gene delivery and expression in planta using chitosan-complexed single-walled carbon nanotube carriers. Nat. Nanotechnol..

[104] Demirer G.S., Zhang H., Goh N.S., Gonzalez-Grandio E., Landry M.P. (2019). Carbon nanotube–mediated DNA delivery without transgene integration in intact plants. Nat. Protoc..

[105] Hou L., Yuan Y., Ren J., Zhang Y., Wang Y., Shan X., Liu Q., Zhang Z. (2017). In vitro and in vivo comparative study of the phototherapy anticancer activity of hyaluronic acid-modified single-walled carbon nanotubes, graphene oxide, and fullerene. J. Nanopart. Res..

[106] Li Y., Li X., Doughty A., West C., Wang L., Zhou F., Nordquist R.E., Chen W.R. (2019). Phototherapy using immunologically modified carbon nanotubes to potentiate checkpoint blockade for metastatic breast cancer. Nanomedicine.

[107] Tondro G.H., Behzadpour N., Keykhaee Z., Akbari N., Sattarahmady N. (2019). Carbon@ polypyrrole nanotubes as a photosensitizer in laser phototherapy of Pseudomonas aeruginosa. Coll. Surf. B.

[108] Xie L., Wang G., Zhou H., Zhang F., Guo Z., Liu C., Zhang X., Zhu L. (2016). Functional long circulating single walled carbon nanotubes for fluorescent/photoacoustic imaging-guided enhanced phototherapy. Biomaterials.

[109] Sobhani Z., Behnam M.A., Emami F., Dehghanian A., Jamhiri I. (2017). Photothermal therapy of melanoma tumor using multiwalled carbon nanotubes. Int. J. Nanomed..

[110] Iannazzo D., Pistone A., Galvagno S., Ferro S., De Luca L., Monforte A.M., Da Ros T., Hadad C., Prato M., Pannecouque C. (2015). Synthesis and anti-HIV activity of carboxylated and drug-conjugated multi-walled carbon nanotubes. Carbon.

[111] Yeh Y.-T., Gulino K., Zhang Y.H., Sabestien A., Chou T.-W., Zhou B., Lin Z., Albert I., Lu H., Swaminathan V. (2020). A rapid and label-free platform for virus capture and identification from clinical samples. Proc. Natl. Acad. Sci. USA.

[112] Sah U., Sharma K., Chaudhri N., Sankar M., Gopinath P. (2018). Antimicrobial photodynamic therapy: Single-walled carbon nanotube (SWCNT)-Porphyrin conjugate for visible light mediated inactivation of Staphylococcus aureus. Coll. Surf. B.

[113] Paquin F., Rivnay J., Salleo A., Stingelin N., Silva C. (2013). Multi-phase semicrystalline microstructures drive exciton dissociation in neat plastic semiconductors. arXiv.

[114] Chaudhari A.A., Jasper S.L., Dosunmu E., Miller M.E., Arnold R.D., Singh S.R., Pillai S. (2015). Novel pegylated silver coated carbon nanotubes kill Salmonella but they are non-toxic to eukaryotic cells. J. Nanobiotechnol..

[115] Khazaee M., Ye D., Majumder A., Baraban L., Opitz J., Cuniberti G. (2016). Non-covalent modified multi-walled carbon nanotubes: Dispersion capabilities and interactions with bacteria. Biomed. Phys. Eng. Exp..

[116] Bhaduri B., Engel M., Polubesova T., Wu W., Xing B. (2018). Dual functionality of an Ag-Fe3O4-carbon nanotube composite material: Catalytic reduction and antibacterial activity. J. Environ. Chem. Eng..

[117] Wang X., Zhou Z., Chen F. (2017). Surface modification of carbon nanotubes with an enhanced antifungal activity for the control of plant fungal pathogen. Materials.

[118] Hao Y., Cao X., Ma C., Zhang Z., Zhao N., Ali A., Hou T., Xiang Z., Zhuang J., Wu S. (2017). Potential applications and antifungal activities of engineered nanomaterials against gray mold disease agent Botrytis cinerea on rose petals. Front. Plant Sci..

[119] Zare-Zardini H., Amiri A., Shanbedi M., Memarpoor-Yazdi M., Assodeh A. (2013). Studying of antifungal activity of functionalized multiwalled carbon nanotubes by microwave-assisted technique. Surf. Interface Anal..

[120] Jawahar N., Surendra E., Krishna K.R. (2015). A review on carbon nanotubes: A novel drug carrier for targeting to cancer cells. J. Pharm. Sci. Res..

[121] Ando Y. (2010). Carbon nanotube: The inside story. J. Nanosci. Nanotechnol..

[122] Basu B., Mehta G.K. (2014). Carbon nanotubes: A promising tool in drug delivery. Int. J. Pharma Biosci..

[123] Arruebo M., Galan M., Navascues N., Tellez C., Marquina C., Ibarra M.R., Santamaria J. (2006). Development of magnetic nanostructured silica-based materials as potential vectors for drug-delivery applications. Chem. Mater..

[124] Varshney K. (2014). Carbon nanotubes: A review on synthesis, properties and applications. Int. J. Eng. Res. Gen. Sci..

[125] Veetil J.V., Ye K. (2009). Tailored carbon nanotubes for tissue engineering applications. Biotechnol. Prog..

[126] Mech L.D. (2014). A Gray Wolf (Canis lupus) delivers live prey to a pup. Can. Field-Nat..

[127] Pradeep Kumar S., Pratibha D., Gowri Shankar N.L., Parthibarajan R., Mastyagiri L., Shankar M. (2012). Pharmaceutical applications of carbon nanotube-mediated drug delivery systems. Int. J. Pharm. Sci. Nanotechnol..

[128] Haddon R.C., Hamon M.A. (2003). Method of Solubilizing Carbon Nanotubes in Organic Solutions. U.S. Patent.

[129] Ono T., Masayoshi E. (2004). Fabrication Methods of Carbon Nanotubes. U.S. Patent.

[130] Shaffer M.S.P., Windle A.H., Brain F.G., Johnson T.M.L., Geng J., Shephard D., Singh C. (2019). Cvd Synthesis of Carbon Nanotubes. EP.

[131] Liu K., Jiang K.-L., Fan S.-S. (2009). Method for Manufacturing Carbon Nanotubes. U.S. Patent.

[132] Dai H., Jiao L. (2012). Narrow Graphene Nanoribbons from Carbon Nanotubes. U.S. Patent.

[133] Moravsky A.P., Loutfy R.O. (2013). Double-Walled Carbon Nanotubes and Methods for Production and Application. U.S. Patent.

[134] Kinloch I., Singh C., Shaffer M.S.P., Koziol K.K.K., Windle A.H. (2017). Method for Producing Carbon Nanotubes and/or Nanofibres. EP.

[135] Noyes D.B. (2018). Methods of Forming Carbon Nanotubes Having a Bimodal Size Distribution. U.S. Patent.

[136] Provine J., Beasley C., Pitner G. (2020). Method for Carbon Nanotube Purification. U.S. Patent.

[137] Hirlekar R., Yamagar M., Garse H., Vij M., Kadam V., Vidyapeeth B. (2009). Carbon nanotubes and its applications: A review. Asian J. Pharm. Clin. Res..

[138] Yang S.-T., Luo J., Zhou Q., Wang H. (2012). Pharmacokinetics, metabolism and toxicity of carbon nanotubes for biomedical purposes. Theranostics.

[139] Wang H., Wang J., Deng X., Sun H., Shi Z., Gu Z., Liu Y., Zhao Y. (2004). Biodistribution of carbon single-wall carbon nanotubes in mice. J. Nanosci. Nanotechnol..

[140] Singh R., Pantarotto D., Lacerda L., Pastorin G., Klumpp C., Prato M., Bianco A., Kostarelos K. (2006). Tissue biodistribution and blood clearance rates of intravenously administered carbon nanotube radiotracers. Proc. Natl. Acad. Sci. USA..

[141] Kagan V.E., Konduru N.V., Feng W., Allen B.L., Conroy J., Volkov Y., Vlasova I.I., Belikova N.A., Yanamala N., Kapralov A. (2010). Carbon nanotubes degraded by neutrophil myeloperoxidase induce less pulmonary inflammation. Nat. Nanotechnol..

[142] Singh G.P.B., Baburao C., Pispati V., Pathipati H., Murthy N., Prassana S.R.V., Rathode B.G. (2012). Carbon nanotubes-A novel drug delivery system. Int. J. Res. Pharm. Chem..

[143] Bobrowska D.M., Olejnik P., Echegoyen L., Plonska-Brzezinska M.E. (2019). Onion-like carbon nanostructures: An overview of bio-applications. Curr. Med. Chem..

[144] Anaya-Plaza E., Shaukat A., Lehtonen I., Kostiainen M.A. (2021). Biomolecule-directed carbon nanotube self-assembly. Adv. Healthc. Mater..

[145] Ahlawat J., Masoudi Asil S., Guillama Barroso G., Nurunnabi M., Narayan M. (2021). Application of carbon nano onions in the biomedical field: Recent advances and challenges. Biomater. Sci..

